# RNF144A-AS1, a TGF-β1- and hypoxia-inducible gene that promotes tumor metastasis and proliferation via targeting the miR-30c-2-3p/LOX axis in gastric cancer

**DOI:** 10.1186/s13578-021-00689-z

**Published:** 2021-09-28

**Authors:** Zengliang Li, Liang Shi, Xiangwei Li, Xiaopeng Wang, Haixiao Wang, Yeliu Liu

**Affiliations:** Department of Gastroenterological Surgery, The Affiliated Huai’an No.1 People’s Hospital of Nanjing Medical University, 1 Huanghe West Road, Huaiyin District, Huai’an, 223300 Jiangsu China

**Keywords:** Gastric cancer, RNF144A-AS1, MiR-30c-2-3p, Lysyl oxidase, Metastasis, Angiogenesis

## Abstract

**Background:**

Although recent molecular analyses have improved our knowledge regarding gastric cancer (GC) biology, the molecular mechanisms that confer metastatic potential to GC remain poorly understood. In this study, we intend to explore the function and characterize the underlying mechanism of long noncoding RNA RNF144A-AS1 in GC metastasis and outgrowth.

**Methods:**

The expression of RNF144A-AS1, miR-30c-2-3p, and Lysyl oxidase (LOX) was detected by quantitative real-time PCR assay. Fluorescence in situ hybridization and subcellular fractionation assay determined the cellular localization of RNF144A-AS1. Cell counting kit 8 assay, transwell assay, and tube formation assay were performed to detect the effect on cell proliferation, migration, invasion, and angiogenesis, respectively. Animal models were also applied to verify the effect on tumor metastasis, outgrowth, and angiogenesis. Bioinformatic analysis, luciferase reporter assay, and RNA immunoprecipitation (RIP) assay explored the interactions among RNF144A-AS1, miR-30c-2-3p, and LOX. Gene regulation was further validated by knockdown of Dicer or mutating the miRNA binding sites on RNF144A-AS1 and LOX 3ʹUTR. Cells were treated with recombinant human TGF-β1 (Transforming Growth Factor β1) to explore the effect of TGF-β1 on RNF144A-AS1. Western blot and immunohistochemistry were used to detect protein expression.

**Results:**

The expression of RNF144A-AS1 was significantly upregulated in GC tissues and was associated with poor prognosis and later-stage diseases. Hypoxia stimulated the expression of RNF144A-AS1 in a HIF-1α-independent manner. Additionally, RNF144A-AS1 was also induced by TGF-β1. Loss and gain of function assays revealed that RNF144A-AS1 promoted tumor metastasis, angiogenesis, and proliferation. Mechanism exploration indicated RNF144A-AS1 served as a microRNA decoy of miR-30c-2-3p to release LOX. Gene Set Enrichment Analysis further suggested LOX and RNF144A-AS1 were enriched in the same gene sets, emphasizing the internal mechanism connection between these two genes.

**Conclusions:**

TGF-β1- and hypoxia-inducible RNF144A-AS1 promoted tumor metastasis, angiogenesis, and proliferation through targeting the miR-30c-2-3p/LOX axis in GC, highlighting the value of the RNF144A-AS1/miR-30c-2-3p/LOX axis in therapeutic interventions of GC.

**Supplementary Information:**

The online version contains supplementary material available at 10.1186/s13578-021-00689-z.

## Introduction

Gastric cancer (GC) is a common neoplastic disease with over 1 million new cases and an estimated 769,000 deaths worldwide in 2020, resulting in a huge socioeconomic burden to the world [1]. Despite the value of multimodal treatment strategies for GC, such as surgery and perioperative chemotherapy, patients diagnosed at advanced stages are prone to locoregional relapse and distant metastases, thence leading to poor survival rates [2, 3]. In 2014, the Cancer Genome Atlas (TCGA) research network proposed four molecularly distinct gastric cancer subtypes and characterized some molecular drivers of GC [4]. However, our understanding of the underlying biology behind the metastatic progression of GC is still poor yet. Therefore, unveiling the mechanism driving metastasis may aid in the development of effective, targeted therapies for this malignancy [5].

In recent years, the roles of long noncoding RNA (lncRNA) in carcinogenesis and malignant processes have attracted accumulating attention [6]. As the name suggested, long noncoding RNAs are a class of non-coding RNAs with a length of more than 200 nucleotides [7]. It is believed the mechanisms of action of lncRNA are associated with its subcellular localization [8]. In general, according to their localization, lncRNAs can be divided into absolutely nuclear-localized type, mainly nuclear-localized type, and largely cytoplasm-localized type. For cytoplasmic lncRNA, it can involve in gene regulation by acting as a decoy or a competing endogenous RNA for miRNAs and proteins [9]. For instance, a study showed lncRNA GCMA (Gastric Cancer metastasis-associated lncRNA) could serve as miRNA sponge via competitively binding miR-124 and miR-34a to upregulate Slug and Snail, thereby promoting GC cell metastasis [10]. Although some lncRNAs that exert tumor-suppressive or oncogenic functions in GC were identified, such as HOTAIR [11], SNHG5 [12], and HNF1A-AS1 [13], the role of plenty of lncRNAs on gastric carcinogenesis are still largely unknown.

Gene Set Enrichment Analysis (GSEA) is a widely used analytical method for interpreting gene expression data [14]. It defines gene sets as groups of genes that share common biological function, chromosomal location, or regulation and then analysis the association of single-gene or groups of genes with these defined gene sets. In this study, lncRNA RNF144A-AS1 was found to be related to the gene set about hypoxia. Hypoxia is a hallmark of the tumor microenvironment, caused by an insufficient blood supply in solid tumors [15]. Diminished oxygen availability increases the expression of hypoxia-inducible factor 1α (HIF-1α) and other pivotal genes, including some aberrantly expressed lncRNAs, which then subsequently promote cancer cell metastasis, angiogenesis, and therapeutic resistance [16, 17]. The extracellular matrix (ECM), which majorly consists of fibrous proteins and proteoglycans, also plays a crucial role in cancer progression [18]. Lysyl oxidase (LOX) is an extracellular cuproenzyme that underpins the fibrotic remodeling of ECM [19]. Under the regulation of HIF-1α, LOX involves in hypoxia-induced remodeling of ECM and malignant progression of carcinomas [20, 21]. In GC, LOX was associated with the epithelial–mesenchymal transition (EMT) of GC cells under hypoxic conditions [22].

In this study, we intend to elucidate the function and characterize the molecular mechanism of lncRNA RNF144A-AS1 in GC. The findings suggest RNF144A-AS1 functions as competing endogenous RNA to bind miR-30c-2-3p and therefore upregulates the expression of LOX.

## Materials and methods

### Patients and specimens

A total of 60 patients diagnosed with GC received radical gastrectomy at the Affiliated Huai'an No.1 People’s Hospital of Nanjing Medical University. The diagnosis of gastric adenocarcinoma was determined by at least two experienced pathologists. All of the patients did not receive any treatment before surgery and had no histories of other malignancy. In addition, eight primary tumor tissues were also collected from patients who received palliative surgery because of distant metastasis. GC tissues and adjacent normal tissues were immediately fetched and placed in liquid nitrogen and were stored at − 80 °C until required. All participants signed written informed consent, and the research was approved by the Ethical Committee on Scientific Research of The Affiliated Huai’an No.1 People’s Hospital of Nanjing Medical University. This study was conducted according to the standards set by the Declaration of Helsinki.

### Cell culture

In this study, GC cell lines such as MKN45, AGS, HGC27, and NCI-N87 were used. Meanwhile, an immortalized normal human gastric mucous epithelium cell line GES-1 was used as control. GES-1, MKN45, HGC27, and NCI-N87 cell lines were cultured in RPMI-1640 medium supplemented with 10% fetal bovine serum and 1% antibiotics (100 U/ml penicillin G and 100 mg/ml streptomycin) (Wisent, Montreal, Canada). AGS cell line was seeded in F-12 K medium with 10% fetal bovine serum and 1% antibiotics, and human embryonic kidney cell line HEK293T and human umbilical vein endothelial cells (HUVECs) were both cultured in Dulbecco’s Modified Eagle’s Medium. For TGF-β1 treatment, recombinant human TGF-β1 (Absin, Shanghai, China) was used at a 5 ng/ml final concentration for 10 days, with TGF-β1 replenishment every 2 days. Cells were incubated in a humidified atmosphere at 37 °C containing 5% CO_2_. All cell lines were obtained from the Cell Bank of the Chinese Academy of Science (Shanghai, China).

### RNA extraction and quantitative real-time PCR (qRT-PCR) analysis

Total RNA was extracted by TRIzol reagent (Invitrogen, MA, USA) from cells and tissues. Complementary DNA (cDNA) was synthesized using a PrimeScript RT reagent kit (TaKaRa, Kyoto, Japan). And miRNAs were reverse transcribed after polyadenylation using Revert Aid First Strand cDNA Synthesis Kit (Thermo Scientific, MA, USA). RT-PCR was performed using SYBR Green premix (Vazyme, Nanjing, China) according to the protocol. GAPDH and U6 were used as the internal control. Specific primers used in this study were summed in Additional file 1: Table S1. Relative RNA abundances were calculated by the standard 2^−ΔΔCt^ method.

### Fluorescence in situ hybridization (FISH)

First, a Cy3-labeled RNF144A-AS1 probe mix was synthesized (RiBo Ltd, Guangzhou, China). In brief, about 20,000 cells were seeded on a 15 mm confocal dish for 24 h. Then the cells were fixed and permeabilized. After pretreatment with the pre-hybridization solution to cells, 20 μM probes were used to hybridize with the cells at 37 °C overnight. Finally, nuclei were stained by DAPI, and the images were captured using a confocal laser scanning microscope (Zeiss LSM5 Live, Oberkochen, German).

### Subcellular fractionation assay

According to the manufacturer’s instructions, nuclear and cytoplasmic RNA separated using the PARIS Kit (Life Technologies, USA).

### Cell transfection and vector construction

Small interfering RNA (siRNA) targeting RNF144A-AS1, LOX, HIF-1α, and Dicer were designed and synthesized, and miRNA mimics and inhibitors for miR-30c-2-3p were also constructed (Genepharma, Shanghai, China). According to the protocol, oligonucleotides (50 nM) were transfected into GC cells using Lipofectamine 3000 (Invitrogen, CA, USA). Then transfected cells were harvested for corresponding analysis and experiments 48 h later. Biologically active short hairpin RNAs (shRNA) targeting RNF144A-AS1 and the sequences of RNF144A-AS1 were both subcloned and amplified into lentiviral expression vectors (Genepharma, Shanghai, China). Stable cell lines were obtained by treating 2 μg/ml puromycin (Sigma-Aldrich, St-Louis, Missouri, USA) for about 3 weeks. The sequences for the above oligonucleotides were shown in Additional file 2: Table S2.

### Transwell migration and invasion assay

Briefly, a total of 20,000 cells were seeded into the upper chamber of the transwell chamber with a pore size of 8 μm (Corning, NY, USA). Meanwhile, the upper and lower chamber were added with serum-free medium or complete medium, respectively. Then, 48 h later, the upper chamber was washed and swabbed, and cells getting through the chamber were stained by 0.1% crystal violet. For invasion, the upper chamber was precoated with Matrigel (BD Bioscience, NJ, USA). Images were collected using a microscope (Ti-E, Nikon, Japan), and cells were counted.

### Wound-healing assay

A wound-healing assay was conducted to examine cellular migration by using a 200 μl sterile pipette tip to mark a linear wound at the bottom of the dish.

### Tube-formation assay

First, 50 μl of Matrigel (BD Bioscience, NJ, USA) was added into a 96-well plate and incubated at 37 °C for 30 min. Then HUVECs were suspended and co-cultured with indicated conditioned medium on the Matrigel. After 4–6 h of incubation, the tubular structures were observed, and images were captured using a bright-field microscope (OLYMPUS CKX41, Japan). Tube formation was quantified by measuring the total length of the tubes using ImageJ software.

### Cell counting kit 8 (CCK8) assay

Cell counting kit 8 (CCK8) was applied to determine the effect on cell proliferation. Following the manufacturer’s instruction (Beyotime, Shanghai, China), cells were plated in 96-well plates (1000 cells/well), and 10 μl of CCK-8 reagent was added to each well. Absorbance was detected spectrophotometrically at 450 nm (MULTISKAN, Thermo scientific, USA).

### Animal experiments

Five to six weeks old female BALB/c nude mice were used in this study. For the lung metastatic model, 1 × 10^6^ of GC cells were directly injected into the tail vein of nude mice. About 5 weeks later, the mice were euthanized, and the lungs were photographed and subjected to hematoxylin and eosin (H&E) staining. For in vivo angiogenesis assay, 2 × 10^6^ of GC cells were mixed with 500 μl Matrigel, and then subcutaneously injected into the flank of nude mice. Two weeks post-transplantation, the mice were sacrificed, and the Matrigel plugs were collected and photographed. Meanwhile, skin vasculature formation adjacent to the plug was also photographed, and indicators such as vessel diameter and the number of branches were observed either [23]. For the orthotopic xenograft model, GC cells (1 × 10^6^/100 μl phosphate‐buffered saline (PBS)) were inoculated subcutaneously into the side armpit of nude mice. Tumor growth was monitored with a caliper every week and calculated with the formula: volume = 0.52 × length × height × width. At 28 days after injection, mice were euthanized. All animal experiments were approved by the Committee on the Ethics of Animal Experiments of the Nanjing Medical University.

### Immunohistochemistry (IHC) analysis

The detailed procedures were discussed previously [24]. Briefly, the tissue slides underwent de-waxed, rehydrated, and epitope retrieval, and then were incubated with primary antibodies: CD31 (ab28364; Abcam), Ki67 (ab15580; Abcam), and LOX (ab174316; Abcam).

### Dual-luciferase reporter assay

The wild-type and mutant RNF144A-AS1 or 3ʹUTR of LOX were amplified and cloned into pGL3-basic luciferase reporter vector (Promega, Madison, Wisconsin, USA) separately. Then, together with miRNA mimics or control, the reporter vectors were transfected into HEK293T cells. 48 h post-transcription, a dual-Luciferase Reporter Assay System (Promega, Madison, Wisconsin, USA) was used to measure the luciferase activity according to the manufacturer’s instructions. Relative luciferase activity was normalized to *Renilla* luciferase.

### RNA immunoprecipitation (RIP) assay

The RIP assay was conducted through a Magna RIPTM RNA-binding protein immunoprecipitation kit (Millipore, Bedford, MA, USA). The coprecipitated RNAs using anti-Ago2 antibody were then extracted and detected by qRT-PCR.

### Bioinformatic analysis

Gene Set Enrichment Analysis was conducted using GSEA software, version 4.1.0. Gene expression data were extracted from the TCGA database and Cancer Cell Line Encyclopedia (CCLE) database. In this study, the chosen gene sets database was h.all.v7.4.symbols.gmt, and the metric for the ranking gene was Pearson in GSEA analysis. Several online tools were applied to predict corresponding targets of RNF144A-AS1 and miR-30c-2-3p, such as RegRNA 2.0, RNA22-HASS, miRDB, DIANA, and Targetscan. Furthermore, survival curves for genes were downloaded from KMplotter.

### Statistics analysis

All statistical analyses were performed on SPSS v19.0 and GraphPad Prism 6. For statistical comparisons, one-way analysis of varisance and the Wilcoxon test was used for comparisons among multiple groups. Comparisons between two groups were analyzed using two-tailed Student’s t-tests. The correlation between RNF144A-AS1 expression and clinicopathological parameters was assessed using the Chi-squared test. The data are expressed as the mean ± standard deviation (SD). All of the experiments in our study were independently performed in triplicate, P < 0.05 was considered statistically significant.

## Results

### Upregulated expression of RNF144A-AS1 predicted dismal prognosis in GC

**Fig. 1:**
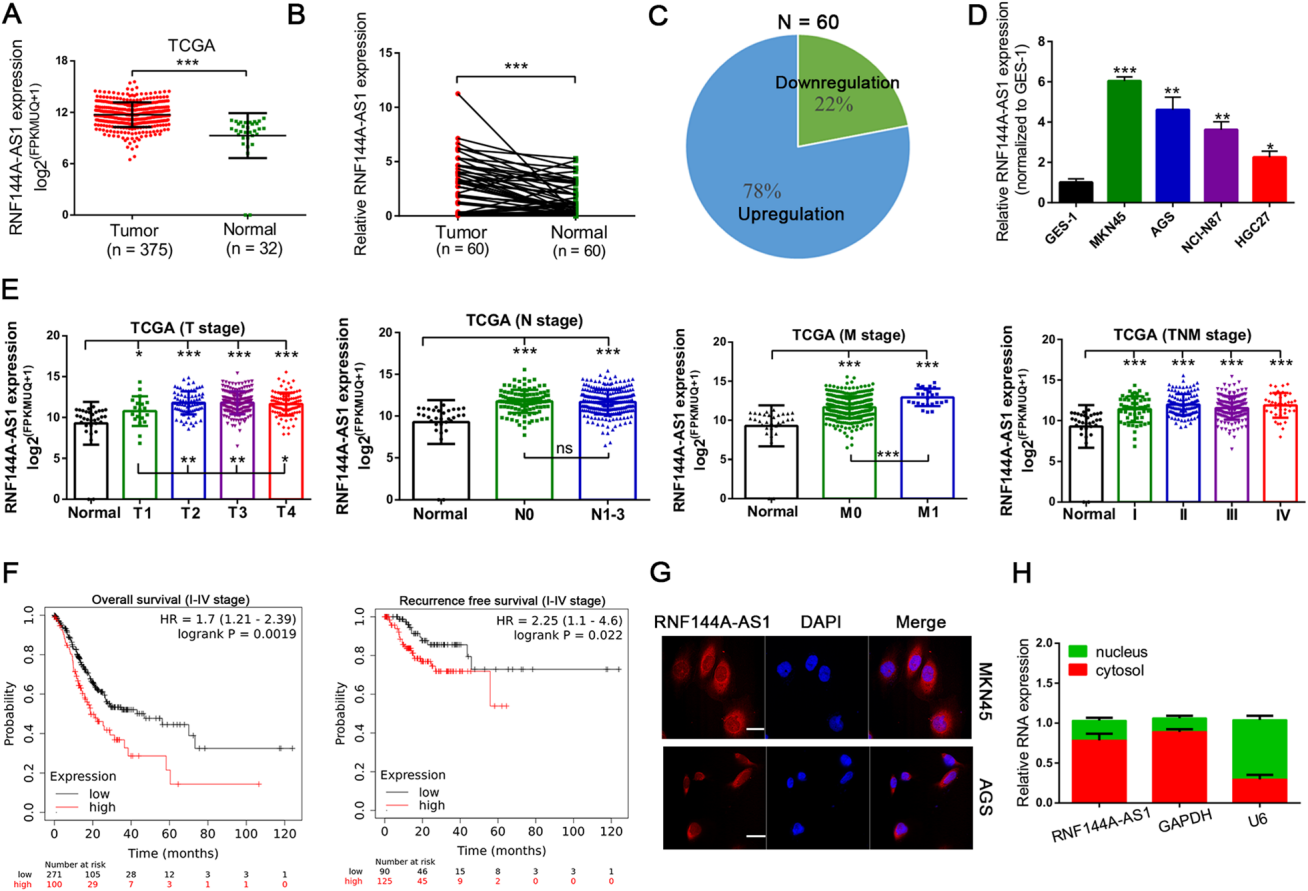
RNF144A-AS1 was upregulated in GC and predicted a poor prognosis. **A** Expression of RNF144A-AS1 in 375 GC tissues and 32 normal gastric tissues from the TCGA database. **B** qRT-PCR analysis of RNF144A-AS1 in 60 paired GC tissues. **C** Presentation of the percentage with upregulated RNF144A-AS1 expression in these 60 paired GC tissues. **D** Expression of RNF144A-AS1 in GC cells as compared with GES-1. **E** Expression patterns of RNF144A-AS1 in tumor tissues relative to normal tissues according to T (Tumor) stage, N (Node) stage, M (Metastasis) stage, and TNM stage from TCGA database. Comparisons were also conducted between groups for T2–3 and T1, N1–3 and N0, M1 and M0. NS, not statistically significant. **F** Kaplan–Meier curves showing overall survival rate and recurrence-free survival rate in GC patients stratified by RNF144A-AS1 expression from KMplotter. *P*-value by Log-rank test. *HR* Hazard ratio. **G** Representative images of the cellular localization of RNF144A-AS1 according to FISH assay. Scale bars = 25 μm. **H** RNF144A-AS1 is largely located in the cytoplasm of GC cells. Error bars, mean ± SD from triplicate samples. *P < 0.05, **P < 0.01, ***P < 0.001 by Student’s t‐test unless otherwise specified

In recent years, using high-throughput sequencing technology, numerous gene expression data have been generated. To identify critical lncRNA implicated in gastric carcinogenesis, integrated analysis of gene expression profiles from the TCGA database was conducted, and the results suggested the expression of RNF144A-AS1 was significantly upregulated in GC tissues (n = 375; Fig. 1A). Furthermore, enhanced expression of RNF144A-AS1 was verified in 60 paired GC tissues, in which 78% of these GC tissues presented with increased expression of RNF144A-AS1 compared with adjacent normal tissues (Fig. 1B, C). Moreover, a higher expression level of RNF144A-AS1 was detected in tumors with distant metastasis than in localized tumors (n = 8) (Additional file 4: Figure S1A). In line with the above findings, GC cells also displayed a higher expression level of RNF144A-AS1 than normal epithelial cell line GES-1 (Fig. 1D). In addition, the clinical relevance of RNF144A-AS1 was also evaluated. From the analysis of the TCGA database, the higher expression level of RNF144A-AS1 was correlated with advanced tumor stages (T stages) and tumor distant metastasis (M stage) (Fig. 1E), as well as linked to poor overall survival rate (HR = 1.70, 95% CI 1.21–2.39) and recurrence-free survival rate (HR = 2.25, 95% CI 1.10–4.60) (Fig. 1F). Beyond this, through categorizing the expression of RNF144A-AS1 as high or low group by using the mean expression level as the cut-off value (n = 26 > mean; n = 34 ≤ mean) in above 60 paired GC tissues, remarkable associations of RNF144A-AS1 with later-stage diseases were discerned, especially with lymph node metastasis (LNM) (*P* = 0.015) (Additional file 3: Table S3). Next, we would like to determine the localization of RNF144A-AS1 in GC cells. Fluorescence in situ hybridization assay and subcellular fraction assay both indicated RNF144A-AS1 was largely cytoplasm-localized (Fig. 1G, H). Meanwhile, the coding potential of RNF144A-AS1 was also investigated. Using online tools like Coding Potential Assessment Tool and ORF finder, the noncoding feature of RNF144A-AS1 was confirmed (Additional file 4: Figure S1B, C).

### RNF144A-AS1 promoted the metastasis, angiogenesis, and proliferation of GC

**Fig. 2:**
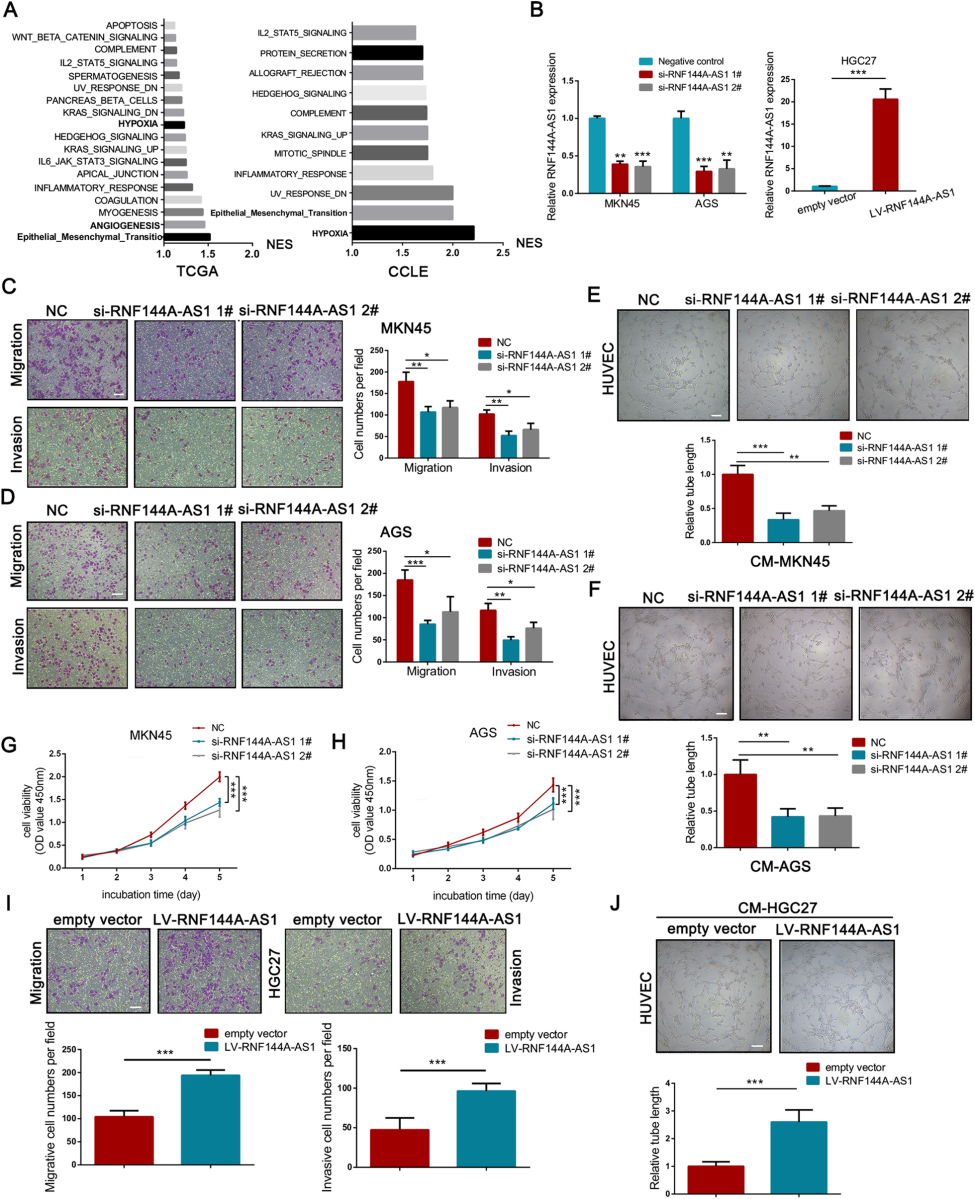
RNF144A-AS1 promoted cellular migration, invasion, proliferation and angiogenesis in GC. **A** GSEA analysis of RNF144A-AS1 based on gene expression data from TCGA database (left panel) and CCLE database (right panel). Normalized Enrichment Score (NES) > 1, Nominal *P*-value < 0.05. **B** Expression level of RNF144A-AS1 in MKN45 and AGS cells transfected with RNF144A-AS1-specific siRNAs (left panel), and in HGC27 cells treated with lentivirus vector containing RNF144A-AS1 coding sequences (right panel). **C**, **D** Detection of the migrative and invasive ability of MKN45 (**C**) and AGS (**D**) cells transfected with RNF144A-AS1-specific siRNAs or negative control (NC) by transwell assays. Scale bars = 100 μm. **E**, **F** Detection of the tube-formation ability of HUVECs after co-cultured with conditioned medium from treated MKN45 (**E**) and AGS (**F**) cells. Scale bars = 200 μm. **G**, **H** Cell growth curves for MKN45 (**G**) and AGS (**H**) cells transfected with RNF144A-AS1-specific siRNAs or negative control. Data were analyzed using two-way ANOVA. **I** Cellular migration and invasion was determined by transwell assays in HGC27 cells transfected with RNF144A-AS1 vector or empty vector. Scale bars = 100 μm. **J** The effect of overexpressing RNF144A-AS1 on the tube-formation ability of HUVECs. Scale bars = 200 μm. Error bars, mean ± SD from triplicate samples. **P < 0.01, ***P < 0.001 by Student’s t‐test unless otherwise specified

**Fig. 3:**
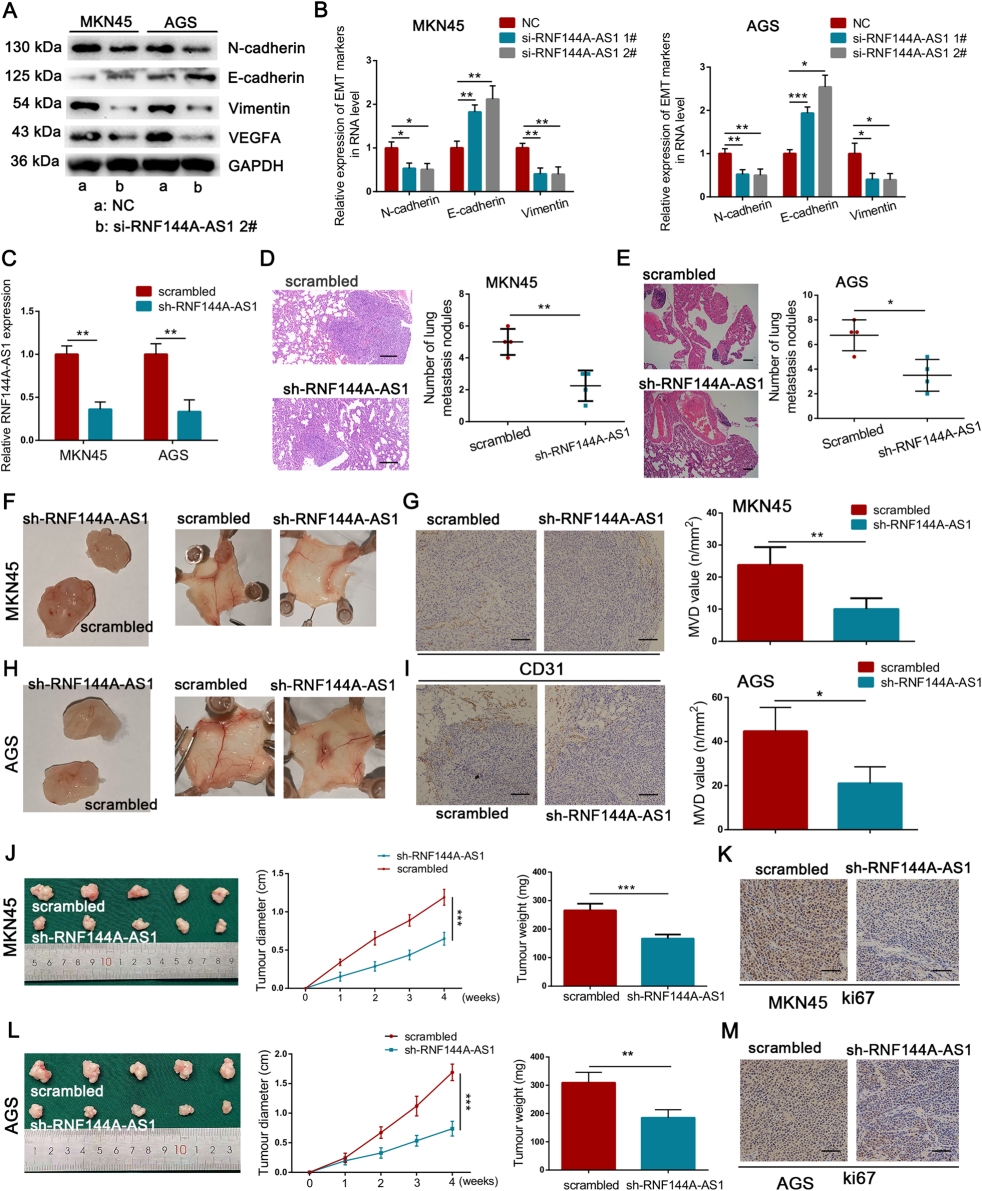
RNF144A-AS1 accelerated tumor metastasis, angiogenesis and growth in vivo. **A** Western bolt analysis of the expression of EMT markers and VEGFA in treated GC cells as indicated. **B** qRT-PCR analysis of the expression of EMT markers in treated GC cells as indicated. **C** Expression level of RNF144A-AS1 in MKN45 and AGS cells transfected with RNF144A-AS1-targeting shRNA or control. **D**, **E** Images of HE staining to lung sections with metastatic sites and the number of lung metastatic nodules to each group using treated MKN45 (**D**) or AGS (**E**) cell lines. Scale bars = 200 μm. **F** Representative images showing Matrigel plugs for RNF144A-AS1 knockdown group and control group (left panel) and representative images of skin vasculature adjoining indicated plugs (right panel). **G** IHC analysis of CD31 in indicated plug sections and the microvascular density (MVD) value was calculated for each group. Scale bars = 100 μm. **H** Representative images of Matrigel plugs and adjoining skin vasculature using treated AGS cells. **I** IHC analysis of CD31 and quantification of the microvascular density from indicated plug sections. Scale bars = 100 μm. **J** and** L** Xenograft tumors from MKN45 (**J**) and AGS (**L**) cells were harvest from nude mice (left panel), and the growth curve (middle panel) and final tumor weight (right panel) were measured. **K** and **M** Images of Ki67 staining for xenograft tumor sections from MKN45 (**K**) or AGS (**M**) cells. Scale bars = 50 μm. Error bars, mean ± SD from triplicate samples. *P < 0.05, **P < 0.01, ***P < 0.001 by Student’s t‐test unless otherwise specified

First, using gene expression data from the TCGA database and CCLE database, GSEA analysis indicated that RNF144A-AS1 was enriched in some key gene sets such as hypoxia, EMT, angiogenesis, apical junction (NES > 1.0, NOM *P*-value < 0.05) (Fig. 2A; Additional file 5: Figure S2A, B). Then, to validate the above predictions in GC cells, the expression of RNF144A-AS1 was downregulated by siRNAs in MKN45 and AGS cells and was overexpressed in HGC27 cells (Fig. 2B). Next, transwell assays showed the migrative and invasive ability of GC cells were seriously harmed when transfected with siRNAs targeting RNF144A-AS1 (Fig. 2C, D). Meanwhile, wound-healing assays further consolidated the inhibition of cellular migration by knockdown of RNF144A-AS1 in MKN45 and AGS cells (Additional file 5: Figure S2C, D). Additionally, conditioned medium obtained from RNF144A-AS1-silenced MKN45 (CM-MKN45) and AGS (CM-AGS) cells progressively decreased the tube-formation rate of HUVECs, suggesting attenuated tumor angiogenesis by the knockdown of RNF144A-AS1 (Fig. 2E, F). Of note, CCK8 assays also suggested that blockade of RNF144A-AS1 impaired the growth of MKN45 and AGS cells (Fig. 2G, H). In contrast, reintroduction of RNF144A-AS1 into HGC27 cells inversely promoted cellular migration, invasion (Fig. 2I), and proliferation (Additional file 5: Fig. S2E). Moreover, conditioned medium derived from HGC27 cells with overexpressed expression of RNF144A-AS1 stimulated the tube-formation rate of HUVECs, as compared with the control group (Fig. 2J). EMT represents one of the cardinal signs of cell invasion and cancer metastasis [25], thus, we then determined the influence of RNF144A-AS1 in this process. As depicted in Fig. 3A and B, silencing RNF144A-AS1 induced the expression of epithelial markers like E-cadherin, accompanied by a dampened expression of mesenchymal markers such as N-cadherin and Vimentin both in protein and RNA levels. Meanwhile, the expression of vascular endothelial growth factor A (VEGFA) was also hampered by the knockdown of RNF144A-AS1 in GC cells, indicating a VEGFA-dependent way for RNF144A-AS1-stimulated angiogenesis. In agreement, it is clear that RNF144A-AS1 contributes to the metastasis, angiogenesis, and proliferation of GC.

To delineate further the functional significances of RNF144A-AS1 in those malignant processes in vivo, we developed multiple animal models. Using RNF144A-AS1-specific short hairpin RNA, the expression of RNF144A-AS1 was diminished (Fig. 3C). First, the lung metastatic model was established by directly injecting treated MKN45 and AGS cells into the tail vein of nude mice. It was evident the number of lung metastatic nodules was sharply reduced in the RNF144A-AS1-silenced group than the control group (Fig. 3D, E). Next, in an attempt to evaluate the effect of RNF144A-AS1 on GC angiogenesis, a Matrigel plug assay was further conducted. Compared with control, downregulation of RNF144A-AS1 markedly decreased the rate of blood vessel formation of Matrigel plugs, as well as reduced skin vascularization adjoining the plug (Fig. 3F, H). In accordance, immunohistochemical analysis of plug sections for angiogenesis marker like platelet endothelial cell adhesion molecule-1 (CD31) also suggested lower blood vessel formation for the RNF144A-AS1-knockdown group (Fig. 3G, I). In addition, nude mice xenograft assays further indicated that depletion of RNF144A-AS1 in GC cells specifically delayed tumor formation and reduced the final tumor weight of subcutaneous tumors when compared with the control group (Fig. 3J, L). Immunohistochemical staining of Ki67, a cell proliferative marker, further substantiated our macroscopic observation (Fig. 3K, M). Hence, these results strongly supported the oncogenic role of RNF144A-AS1 in GC outgrowth, metastasis, and angiogenesis.

### RNF144A-AS1 served as a miRNA decoy for miR-30c-2-3p

**Fig. 4:**
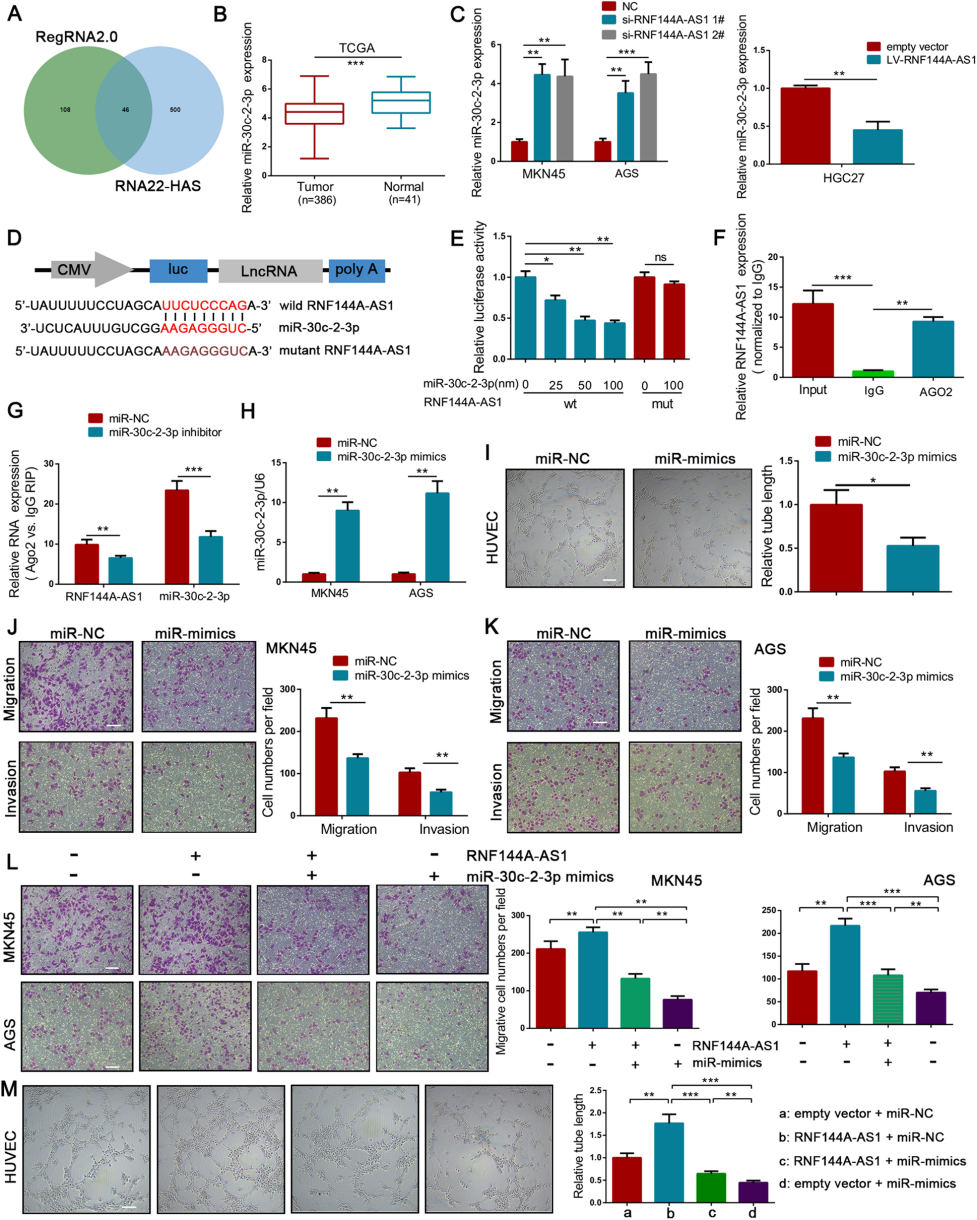
RNF144A-AS1 required miR-30c-2-3p to perform its function. **A** Venn diagram showing potential binding miRNAs for RNF144A-AS1. **B** Expression of miR-30c-2-3p in 386 GC tissues and 41 normal gastric tissues from TCGA database. **C** qRT-PCR analysis shown that the expression of miR-30c-2-3p was negatively regulated by RNF144A-AS1. **D** Construction of luciferase reporter vectors that contain RNF144A-AS1 sequences with or without mutated miR-30c-2-3p binding sites. **E** Luciferase activity was measured in HEK293T cells co-transfected with increasing concentration of miR-30c-2-3p mimics and indicated vectors. **F** qRT-PCR analysis of immunoprecipitated RNF144A-AS1 transcripts in Ago2 relative to IgG immunoprecipitates. **G** Fold enrichment of miR-30c-2-3p and RNF144A-AS1 in immunoprecipitates from cells treated with miRNA inhibitor or control. **H** Expression of miR-30c-2-3p in GC cells transfected with miRNA mimics or control. **I** Tube-formation ability of HUVECs co-cultured with indicated conditioned medium. Scale bars = 200 μm. **J**, **K** Effects on cellular migration and invasion by transfecting miR-30c-2-3p mimics or control into MKN45 (**J**) and AGS (**K**) cells. Scale bars = 100 μm. **L**, **M** Effects of co-transfection of miR-30c-2-3p mimics and RNF144A-AS1 vector on cellular migration (**L**) and tube-formation (**M**). Error bars, mean ± SD from triplicate samples. *P < 0.05, **P < 0.01, ***P < 0.001 by Student’s t‐test unless otherwise specified

Having established that RNF144A-AS1 acts as an oncogene in GC, we next aimed to determine the underlying mechanism. Given the cytoplasmic localization of RNF144A-AS1 in GC cells, online tools such as RegRNA2.0 and RNA22-HAS were applied to predict the potential binding sites of miRNAs to RNF144A-AS1 (Fig. 4A). Because of the relatively low expression level in GC tissues from the TCGA database, miR-30c-2-3p and miR-139-3p were chosen for further research (Fig. 4B; Additional file 6: Figure S3A, B). However, GC cells transfected with RNF144A-AS1-specific siRNA only exhibited upregulated expression of miR-30c-2-3p, but not miR-139-3p (Fig. 4C; Additional file 6: Figure S3C). Consistent with above findings, HGC27 cells transfected with RNF144A-AS1 vector restricted the expression of miR-30c-2-3p (Fig. 4C). Thus, we then exclusively explored the relationship between miR-30c-2-3p and RNF144A-AS1. To validate the interaction, the binding sequences of miR-30c-2-3p to RNF144A-AS1 were mutated and engineered into a luciferase reporter vector (Fig. 4D). As showed in Fig. 4E, the luciferase activity was efficiently reduced following co-transfection with increasing concentration of miR-30c-2-3p and a reporter vector carrying the wild-type sequence of RNF144A-AS1 into HEK293T cells. Nevertheless, mutation of miR-30c-2-3p binding sites abolished the suppressive effect of miR-30c-2-3p mimics on RNF144A-AS1-driven luciferase activity (Fig. 4E). MiRNAs are involved in the formation of RNA-silencing complex with Argonaute 2 (Ago2) and can guide the complex to bind targeted genes, thereby reducing the expression of downstream targets [26]. In this regard, we then attempted to ensure the influence of RNF144A-AS1 on the miR-30c-2-3p-dependent RNA-silencing complex. As expected, using an anti-Ago2 antibody, transcripts of RNF144A-AS1 were efficiently immunoprecipitated from AGS cells (Fig. 4F). Moreover, the qRT-PCR analysis showed compared with the control group, both the expression level of RNF144A-AS1 and miR-30c-2-3p were drastically decreased in the immunoprecipitation purified from AGS cells transfected with miR-30c-2-3p inhibitor (Fig. 4G). Additionally, correlation analysis of 60 GC tissues indicated an inverse correlation between RNF144A-AS1 and miR-30c-2-3p (*R*^2^ = 0.2448, *P* < 0.001) (Additional file 6: Figure S3D). Based on these findings, we concluded that lncRNA RNF144A-AS1 could bind with miR-30c-2-3p and negatively regulate its expression in GC.

MiR-30c-2-3p is a well-defined tumor suppressor that can target a variety of cancer-associated genes, yet its function in GC is still poorly understood [27]. As suggested in Fig. 4H, significantly upregulated expression of miR-30c-2-3p was observed in GC cells transfected with miRNA mimics, while restricted expression of miR-30c-2-3p was discerned by miR-30c-2-3p-specific inhibitor (Additional file 6: Figure S3E). First, the tube-formation ability of HUVECs was severely inhibited after co-cultured with conditioned medium derived from AGS cells treated with miR-30c-2-3p mimics (Fig. 4I). Meanwhile, transwell assays with or without Matrigel coating showed overexpression of miR-30c-2-3p resulted in less migration and invasion than that observed in control cells (Fig. 4J, K). Remarkably, the inhibitory effect of miR-30c-2-3p on EMT was detected either, characterized by increased expression of E-cadherin and decreased expression of N-cadherin and Vimentin after overexpression of miR-30c-2-3p (Additional file 6: Figure S3F). Moreover, the expression level of VEGFA was potently abolished by miR-30c-2-3p either (Additional file 6: Figure S3F). Thus, these results indicated that a low expression level of miR-30c-2-3p may be indispensable for GC aggressiveness.

To determine whether miR-30c-2-3p mediates the promotive effect of RNF144A-AS1 on GC, we co-transfected miR-30c-2-3p mimics and RNF144A-AS1 vector into GC cells. First, transwell assays suggested RNF144A-AS1-mediated promotions on cellular migration and invasion were markedly reversed by miR-30c-2-3p mimics (Fig. 4L; Additional file 6: Figure S3G). Tube-formation assays also suggested miR-30c-2-3p could phenocopy RNF144A-AS-driven angiogenesis (Fig. 4M). In the same direction, ectopic miR-30c-2-3p rescued the effects of RNF144A-AS1 on cell proliferation (Additional file 6: Figure S3H, I). In summary, we found RNF144A-AS1 promotes tumor metastasis, angiogenesis, and proliferation by competitively binding miR-30c-2-3p.

### LOX was determined as the target of miR-30c-2-3p

**Fig. 5:**
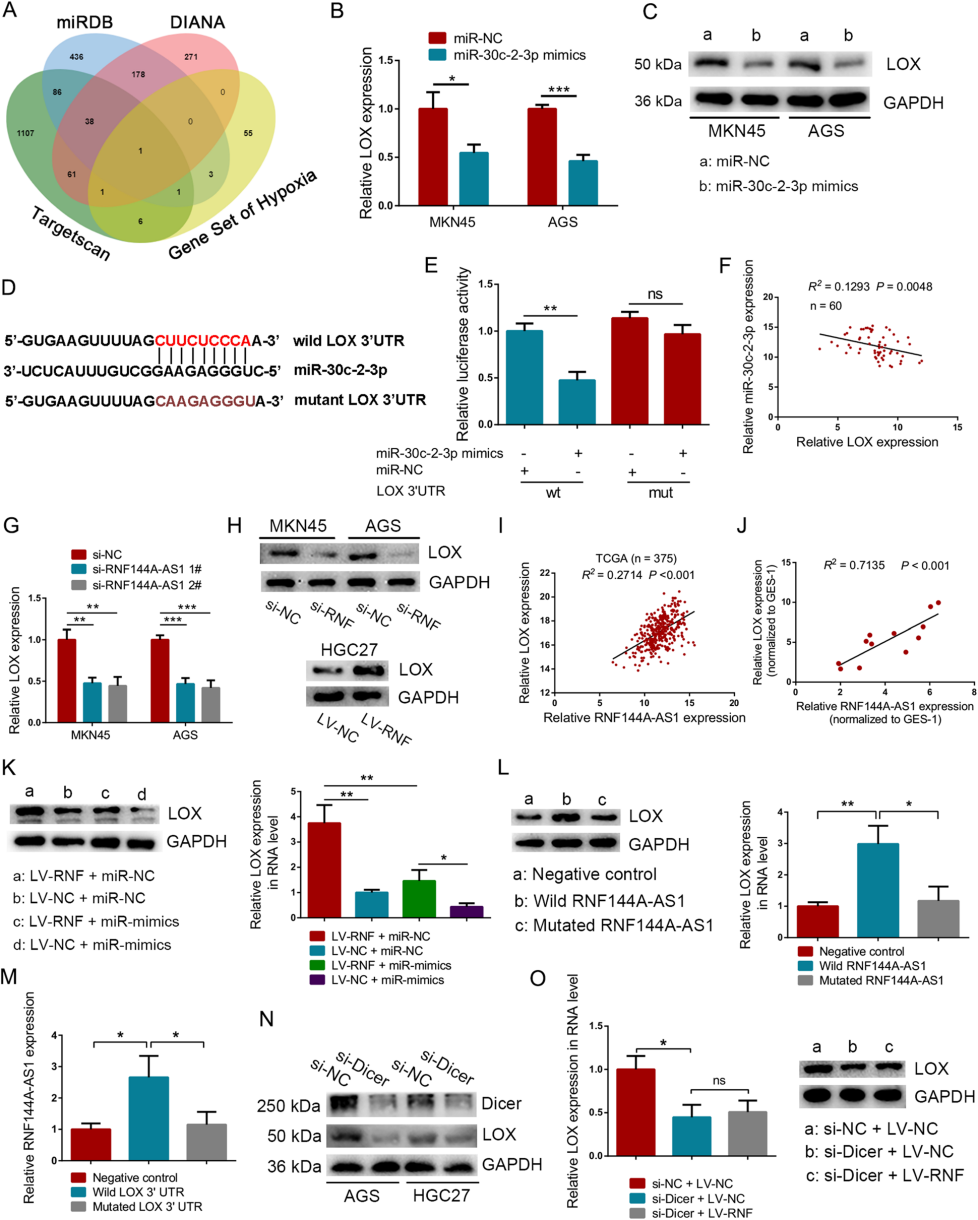
LOX was determined as the target of miR-30c-2-3p. **A** Venn diagram showing predicated target of miR-30c-2-3p by intersecting four datasets. **B**, **C** Expression of LOX in RNA level (**B**) and protein level (**C**) as indicated. **D** The binding sites of miR-30c-2-3p to LOX 3′UTR were mutated and engineered into a luciferase reporter vector. **E** Luciferase activity for each group as indicated. NS represents not significant. **F** Pearson correlation analysis between LOX and miR-30c-2-3p in 60 GC tissues. **G** qRT-PCR analysis of LOX expression after downregulating RNF144A-AS1. **H** Western blot analysis of LOX expression in MKN45 and AGS cells transfected with RNF144A-AS1-specific siRNA and in HGC27 cells transduced with RNF144A-AS1 vector. Abbreviation of RNF represents RNF144A-AS1. **I**, **J** Pearson correlation analysis between LOX and RNF144A-AS1 in 375 GC tissues from the TCGA database (**I**) and in GC cell lines (**J**). **K** Analysis of LOX expression in protein and RNA levels from treated HGC27 cells. **L** Detection of LOX in HGC27 cells transduced with wild-type RNF144A-AS1 expression plasmids, miR-30c-2-3p binding sites-mutated RNF144A-AS1 plasmids or control. **M** Quantitative analysis of RNF144A-AS1 expression as indicated. **N** Expression level of Dicer and LOX in GC cells treated with Dicer targeting-siRNA. **O** Detection of LOX in HGC27 cells co-transfected with RNF144A-AS1 vector and Dicer targeting-siRNA. Error bars, mean ± SD from triplicate samples. *P < 0.05, **P < 0.01, ***P < 0.001 by Student’s t‐test unless otherwise specified

The competing endogenous RNA (ceRNA) model demands the interactions among lncRNA, miRNA, and downstream targets. Here, LOX was predicted as the target of miR-30c-2-3p through intersecting four datasets, including the gene set about hypoxia enriched by RNF144A-AS1 and target sets generated by miRDB, DIANA, and Targetscan (Fig. 5A). Notably, GC cells transfected with miR-30c-2-3p mimics exhibited lower expression of LOX both in RNA and protein level, compared with the control group (Fig. 5B, C). Meanwhile, downregulating of miR-30c-2-3p induced elevated expression of LOX either (Additional file 7: Figure S4A, B). Moreover, the 3′ UTR of LOX was fused into a luciferase reporter vector, and another reporter vector containing mutated miR-30c-2-3p binding sites was also constructed (Fig. 5D). Then these vectors together with miR-30c-2-3p mimics were co-transfected into HEK293T cells, respectively. As expected, mutating miR-30c-2-3p seed sequences in LOX 3′ UTR was sufficient to abolish miR-30c-2-3p-dependent regulation of luciferase activity (Fig. 5E). On the other hand, an inverse correlation between LOX and miR-30c-2-3p was detected in 60 GC tissues (*R*^2^ = 0.1293, *P* = 0.0048) (Fig. 5F). In this regard, it is evident that LOX is the target of miR-30c-2-3p. However, it is also important to ensure the regulation of RNF144A-AS1 to LOX. First, depletion of RNF144A-AS1 drastically suppressed the expression of LOX in MKN45 and AGS cells, while artificial expression of RNF144A-AS1 significantly boosted LOX expression in HGC27 cells (Fig. 5G, H). Using expression data of 375 GC tissues from the TCGA database, we detected a positive correlation between RNF144A-AS1 and LOX either (*R*^2^ = 0.2714, *P* < 0.001), which was consistent with the result from GC cells (*R*^2^ = 0.7135, *P* < 0.001) (Fig. 5I, J). Importantly, restoring the expression of miR-30c-2-3p progressively rescued RNF144A-AS1-induced upregulation of LOX in HGC27 cells (Fig. 5K). Meanwhile, co-transfection with miR-30c-2-3p inhibitor also rescued the expression of LOX in MKN45 cells transfected with siRNA against RNF144A-AS1 (Additional file 7: Figure S4C). For further confirmation, we constructed expression plasmids containing the sequence of RNF144A-AS1, which contains wild-type (Wild) or mutated miR-30c-2-3p binding sites (Mutated). We found overexpression of RNF144A-AS1 drastically enhanced the expression of LOX but not empty plasmid or mutant plasmid (Fig. 5L). Furthermore, in a similar manner, mutated LOX 3ʹUTR or empty plasmid could not vary the expression of RNF144A-AS1 except for the wild type with the binding sites of miR-30c-2-3p (Fig. 5M). Hence, the above findings confirmed the competition to miR-30c-2-3p between LOX and RNF144A-AS1. Previous researches have established the fundamental role of Dicer in miRNA biogenesis [28]. Therefore, we intend to further testify that miR-30c-2-3p acts as an intermediary between RNF144A-AS1 and LOX by knockdown of Dicer. First, silencing of Dicer successfully inhibited the expression of LOX, emphasizing the important role of miRNAs in the regulation of LOX (Fig. 5N). Then, as expected, when we co-transfected Dicer targeting-siRNA and RNF144A-AS1 vector into HGC27 cells, the expression of LOX did not make a difference compared with the Dicer knockdown group (Fig. 5O). Therefore, these results supported the ceRNA model among RNF144A-AS1, miR-30c-2-3p, and LOX.

### LOX improved the aggressiveness of GC

**Fig. 6:**
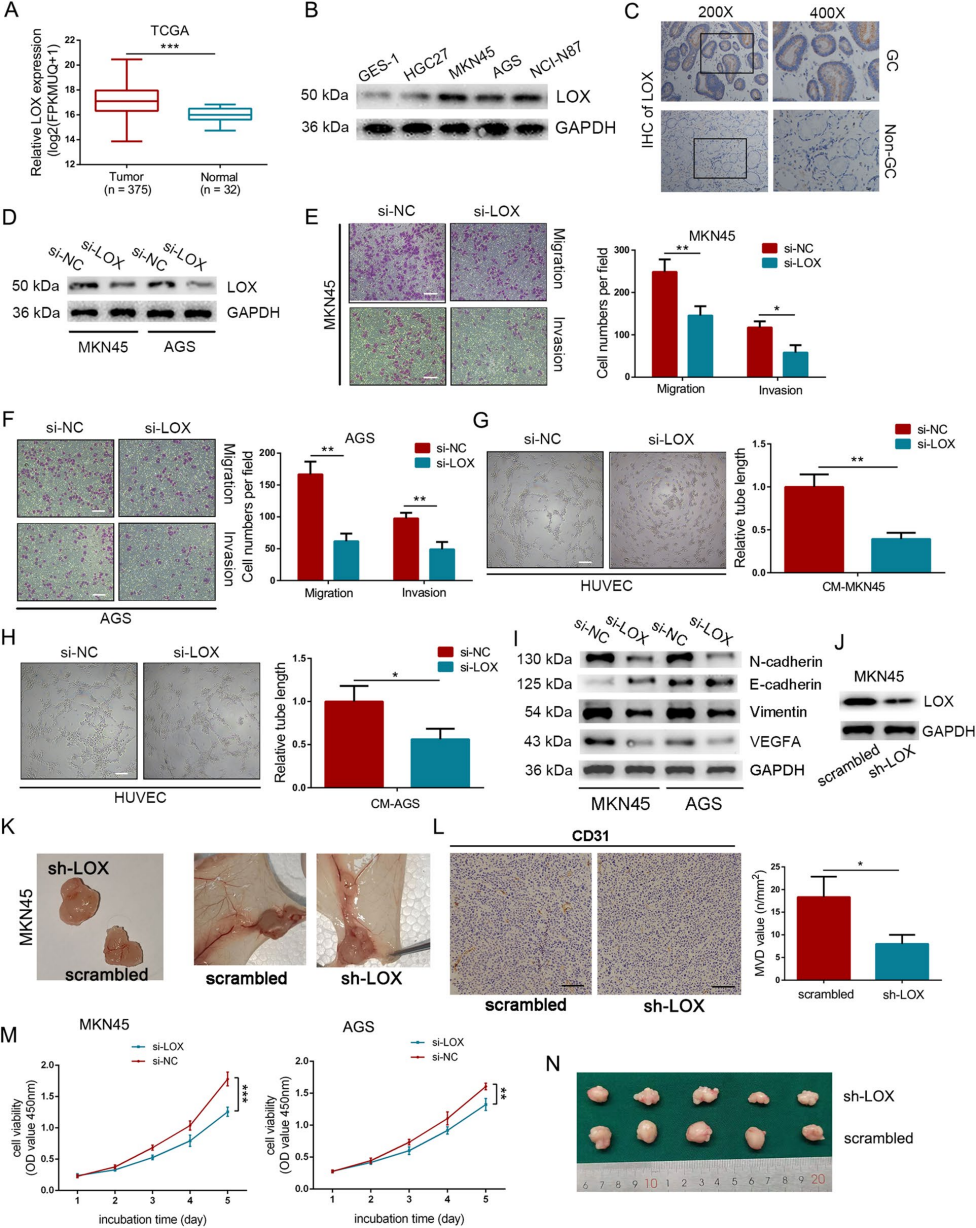
LOX improved the aggressiveness of GC. **A** Expression level of LOX in GC tissues from the TCGA database. **B** Expression level of LOX in GC cells and normal epithelial cell line GES-1. **C** IHC analysis of LOX in GC tissue and adjacent normal tissue. **D** Western blot analysis of the efficiency of LOX-specific siRNA in GC cells. **E, F** Effect on cellular migration and invasion by downregulating LOX in MKN45 (**E**) and AGS cells (**F**). Scale bar = 100 μm. **G**, **H** Silencing LOX in MKN45 (**G**) and AGS (**H**) cells inhibited the tube-formation rate of HUVECs. Scale bar = 200 μm. **I** Western blot analysis of the expression of EMT markers and VEGFA in LOX-knockdown group or control group. **J** LOX-targeting shRNA was used. **K** Representative images of blood vessel formation to plugs (left panel) and skin vasculatures around the plugs (right panel). **L** IHC staining of CD31 to plug sections as indicated and the microvascular density (MVD) values for each group. Scale bars = 100 μm. **M** Detection of cell growth by CCK8 assays in treated MKN45 and AGS cells. Data were analyzed using two-way ANOVA. **N** Representative images of gross appearance of subcutaneous tumors from nude mice. Error bars, mean ± SD from triplicate samples. *P < 0.05, **P < 0.01, ***P < 0.001 by Student’s t‐test unless otherwise specified

HIF-1α-induced expression of LOX typically causes stiffening of the ECM in cancer, thereby allowing cancer cells to easily metastasize [29]. In our study, we delineate further the function of LOX in GC. Indeed, predominant overexpression of LOX was observed in GC tissues and cells (Fig. 6A–C). In addition, a high expression level of LOX was strongly associated with poor overall survival, particularly in patients diagnosed at advanced stages (Additional file 7: Figure S4D). Then, the expression of LOX was efficiently knocked down by siRNA in GC cells (Fig. 6D). As illustrated, depletion of LOX drastically inhibited the migrative and inhibitive ability of GC cells (Fig. 6E, F). Tube-formation assay also suggested dampened angiogenetic ability of HUVECs after co-cultured with LOX-silenced conditioned medium, as compared with control (Fig. 6G, H). Furthermore, Western blot analysis indicated knockdown of LOX inhibited EMT process on GC cells and the expression of VEGFA (Fig. 6I). Remarkably, in vivo observations revealed reduced blood vessel formation of plugs, as well as decreased skin vascularization in the LOX knockdown group (Fig. 6J, K). And IHC staining of CD31 further emphasized the attenuation of angiogenesis in Matrigel mixed with GC cells transfected by LOX siRNA (Fig. 6L). A similar phenomenon was also detected in cell proliferation, in which downregulating of LOX inhibited cell growth and tumor formation both in vivo and in vitro (Fig. 6M, N). Therefore, it is reasonable to believe that LOX could promote tumor metastasis, angiogenesis, and proliferation in GC.

### LOX was required for the function of miR-30c-2-3p in GC

**Fig. 7:**
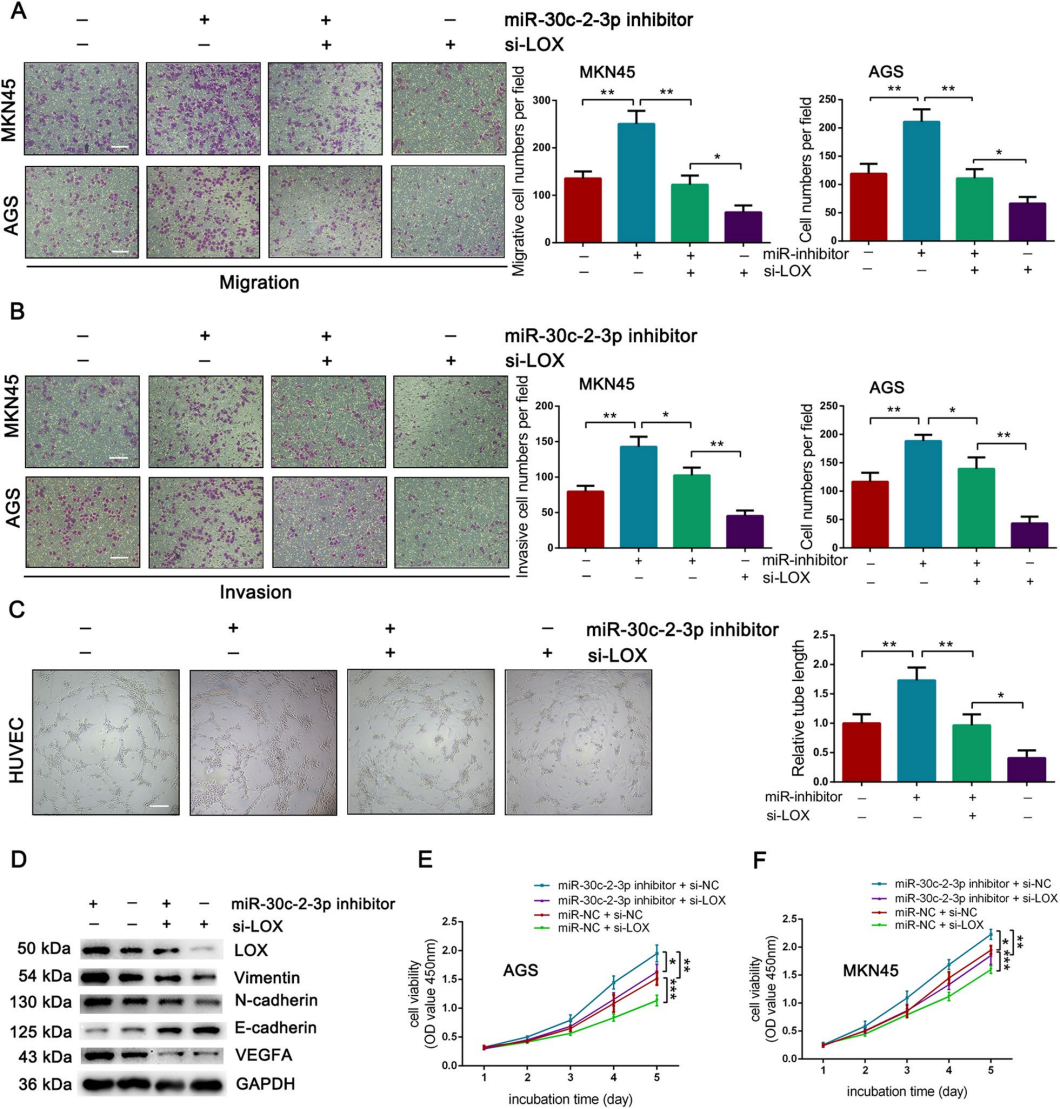
LOX was necessary for the function of miR-30c-2-3p. **A**, **B** Effect on cellular migration (**A**) and invasion (**B**) by co-transfecting miR-30c-2-3p inhibitor and LOX siRNA into MKN45 and AGS cells. Scale bar = 100 μm. **C** Tube-formation assays for HUVECs co-cultured with conditioned medium filtrated from treated GC cells. Scale bar = 200 μm. **D** Western blot analysis of the expression of LOX, EMT markers, VEGFA in AGS cells co-transfected with miR-30c-2-3p inhibitor and LOX-specific siRNA. **E**, **F** CCK8 assays for AGS (**E**) and MKN45 (**F**) cells as indicated. Data were analyzed using two-way ANOVA. Error bars, mean ± SD from triplicate samples. *P < 0.05, **P < 0.01 by Student’s t‐test unless otherwise specified

To evaluate the role of LOX in mediating the function of miR-30c-2-3p, we co-transfected LOX-specific siRNA and miR-30c-2-3p inhibitor into GC cells. First, Transwell assays indicated that induced promotion on cellular invasion and migration by miR-30c-2-3p inhibitor was mitigated through downregulating of LOX (Fig. 7A, B). And an analogous mode was detected in tube-formation assays (Fig. 7C). Notably, Western blot analysis showed the same influence on the expression of LOX, EMT-markers, and VEGFA, which miR-30c-2-3p inhibitor rescued the suppression of these genes in the LOX-knockdown group (Fig. 7D). Meanwhile, the CCK8 assays indicated that miR-30c-2-3p inhibitor could rescue the impaired cell growth in the LOX-silencing group (Fig. 7E, F). Therefore, the function of miR-30c-2-3p is orchestrated by its negative regulation of LOX.

### RNF144A-AS1 was a TGF-β- and hypoxia-inducible gene in GC

**Fig. 8:**
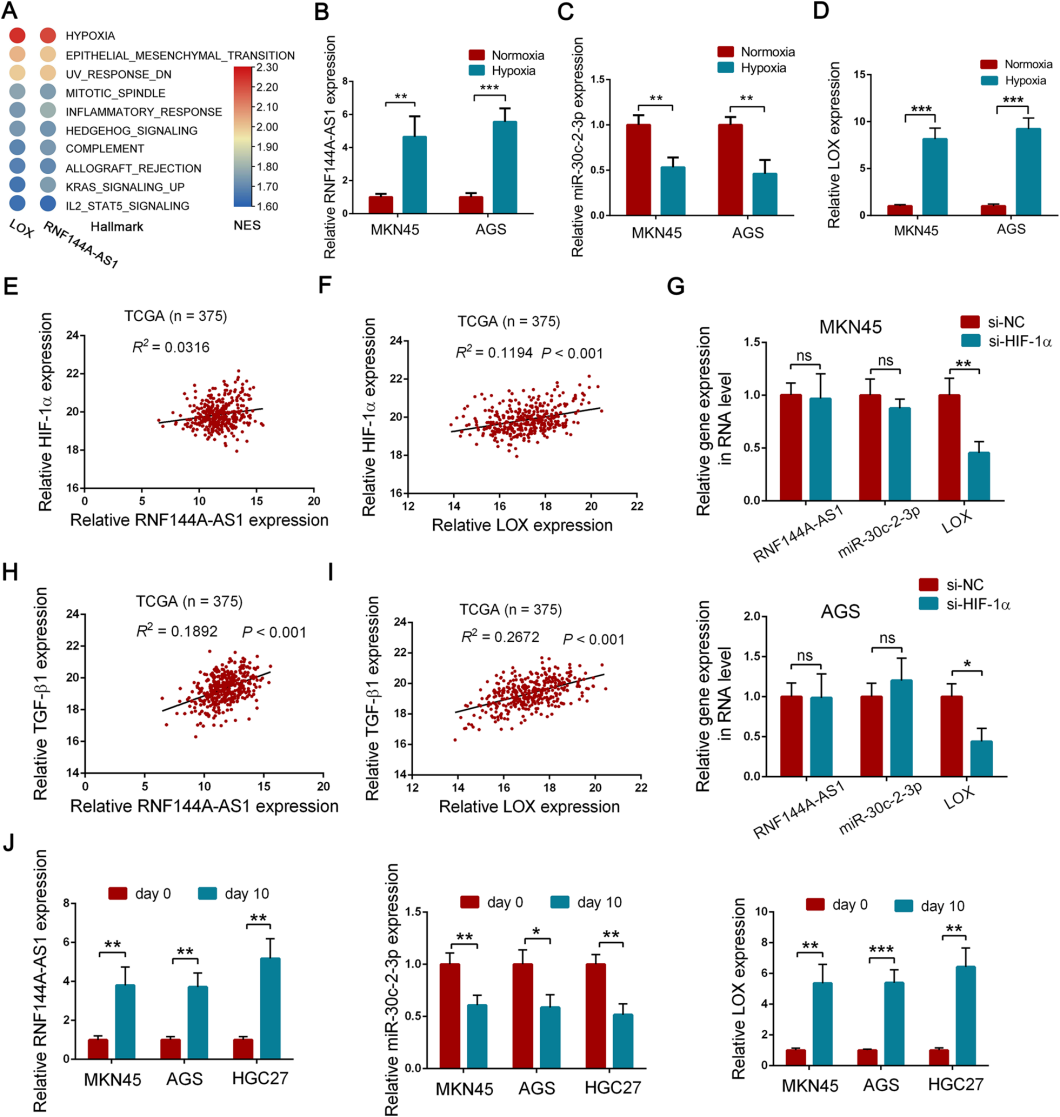
RNF144A-AS1 was a TGF-β- and hypoxia-inducible gene in GC. **A** Heat map presentation of the results generated by GSEA analysis based on the expression of LOX and RNF144A-AS1 from the CCLE database. The red shades represent high NES scores, and the blue shades represent low NES scores. **B–D** qRT-PCR analysis of RNF144A-AS1 (**B**), miR-30c-2-3p (**C**), and LOX (**D**) expression under hypoxic conditions. **E**, **F** Pearson correlation analysis of RNF144A-AS1 (**E**) and LOX (**F**) with HIF-1α in 375 GC tissues from the TCGA database. **G** Expression level of RNF144A-AS1, miR-30c-2-3p, and LOX in HIF-1α-knockdown group. **H**, **I** Pearson correlation analysis of RNF144A-AS1 (**H**) and LOX (**I**) with TGF-β1. **J** Expression level of RNF144A-AS1, miR-30c-2-3p, and LOX in GC cells treated with recombinant human TGF-β1 or not. Error bars, mean ± SD from triplicate samples. *P < 0.05, **P < 0.01, ***P < 0.001 by Student’s t‐test unless otherwise specified

Since RNF144A-AS1 tightly regulates the expression of LOX through targeting miR-30c-2-3p, we, therefore, conducted GSEA analysis on LOX to evaluate whether LOX and RNF144A-AS1 are jointly involved in certain cellular phenotypes or signaling pathways. Intriguingly, LOX and RNF144A-AS1 were enriched in the same gene sets with relatively similar enrichment scores using gene expression data from the CCLE database, which indicates RNF144A-AS1 functions synchronously with LOX in GC (Fig. 8A; Additional file 7: Figure S4E, F). Moreover, we then detected the expression of RNF144A-AS1, miR-30c-2-3p, and LOX under hypoxic conditions. It showed the expression of RNF144A-AS1 and LOX was significantly elevated under the hypoxic circumstance, while the expression of miR-30c-2-3p was greatly downregulated (Fig. 8B–D). Moreover, compelling evidence has shown the reciprocal interplay between LOX, HIF-1α, and hypoxia. Therefore, we decided to explore the potential interaction of RNF144A-AS1 and HIF-1α. However, through the correlation analysis among RNF144A-AS1, LOX, and HIF-1α, we did not find a relationship between RNF144A-AS1 and HIF-1α (Fig. 8E, F). Meanwhile, knockdown of HIF-1α did not influence the expression of RNF144A-AS1 but LOX (Fig. 8G; Additional file 8: Figure S5A). Together, these experiments revealed that hypoxia stimulated RNF144A-AS1 expression in a HIF-1α-independent manner.

Notably, a plethora of studies demonstrates that TGF-β is also a strong promoter of EMT and is significantly involved in tumor metastasis [30]. Thus, we wondering whether TGF-β signaling is one of the upstream activators of the RNF144A-AS1-miR-30c-2-3p-LOX axis. First of all, GSEA analysis indicated a positive correlation of LOX expression and TGF-β signaling, suggesting a potential interaction between them (Additional file 8: Figure S5B, C). Then, through exploring the TCGA database, we detected that the expression of LOX and RNF144A-AS1 were significantly associated with TGF-β1, the main member of the TGF-β family (*R*^2^ = 0.1892 for RNF144A-AS1; *R*^2^ = 0.2672 for LOX) (Fig. 8H, I). To further delineate the role of TGF-β1 on the regulation of RNF144A-AS1 and LOX, GC cells were treated with recombinant human TGF-β1. Compared with day 0, the expression of RNF144A-AS1 and LOX both increased in all GC cell lines after treatment for ten days, whereas the expression of miR-30c-2-3p was suppressed (Fig. 8J; Additional file 8: Figure S5D). Interestingly, a higher fold-change of LOX, rather than RNF144A-AS1, was discerned, indicating that TGF-β1 manipulates LOX expression in multiway. Thus, we concluded that RNF144A-AS1 was induced in a TGF-β1-dependent pathway.

## Discussion

A wealth of literature has discussed the significance of lncRNA on cancer development and progression [31], highlighting the feasibility of lncRNAs as diagnostic biomarkers (i.e., lncRNA-UEGC1 [32], lncRNA-HOTTIP [33]), prognostic indicators (i.e., LINC00346 [34]), and therapeutic targets for GC [35]. In this study, we proposed a critical lncRNA, RNF144A-AS1, that promoted the metastasis, angiogenesis, and proliferation of gastric cancer. However, the understanding of RNF144A-AS1 is deficient yet. RNF144A-AS1, also named as Glycosaminoglycan Regulatory Associated Long Non-coding RNA (GRASLND), was first identified as a regulator of mesenchymal stem cell chondrogenesis [36]. In bladder cancer (BCa), it was reported that an RNF144A-AS1-based nomogram could effectively predict the prognosis of patients with BCa, and RNF144A-AS1 could promote proliferation, migration, and invasion of bladder cancer cells [37, 38]. Consistent with above researches, our study also detected an increased expression of RNF144A-AS1 in GC tissues and also suggested strong associations of RNF144A-AS1 with poor prognosis and later-stage diseases in patients with GC. Furthermore, functional assays proved the promotive effect of RNF144A-AS1 on GC metastasis, angiogenesis, and cell growth. Thus, our results demonstrated an oncogenic role of RNF144A-AS1 on the malignant processes of GC.

The ceRNA model is a characteristic way for lncRNA to involve in gene regulation, which refers to the fact that lncRNA competitively binds to miRNAs. In the present study, RNF144A-AS1 was suggested to act as ceRNA by binding miR-30c-2-3p. In the human genome, pre-miR-30c engenders two mature miRNA, miR-30c-5p (guide strand) and miR-30c-2-3p (passenger strand), which both are believed to be tumor suppressors [27]. For miR-30c-2-3p, its repression enhances HIF-2α expression and could promote cellular proliferation, angiogenesis, and xenograft tumor growth in human clear cell renal cell carcinomas [39]. In GC, miR-30c-2-3p could inhibit cellular growth by targeting the RAB31-GLI1 axis [40]. Here, we found that miR-30c-2-3p repressed cellular proliferation and invasion as well as angiogenesis and metastasis of GC. Of note, most lncRNAs exert their function by cooperating with various proteins [41]. Indeed, RNF144A-AS1 could cooperate with EIF2AK2 to inhibit the expression of IFN-γ revealed by Huynh et al. [36]. Similarly, in GC, RNF144A-AS1 may interact with RNA-binding protein to influence gene expression or act as a cofactor for transcriptional factors and chromatin modifiers to participate in gene regulation, considering the portion of nuclear localization of it [42, 43].

The LOX family, with five paralogs: LOX and LOX-like 1–4 (LOXL 1–4), play a vital role in catalyzing the cross-linkage of collagen and elastin and thus are involved in the formation of primary tumor and the establishment of metastases [44]. In GC, LOX was proved as an EMT promotor under hypoxic conditions [22]. For the liver metastasis of GC, cancer-associated fibroblasts-derived LOX facilitates tumor cell proliferation and outgrowth at the metastatic niche [45]. In addition, LOX was also recognized as essential for angiogenesis by promoting the expression and secretion of VEGF [46–48]. Accordingly, our experimental results also indicated the induction of VEGFA by forcing the expression of LOX. Importantly, LOX is recognized as a core factor of the hypoxic tumor microenvironment, such as through forming a feedback loop with HIF-1α [21]. In this study, LOX was verified as the downstream target of miR-30c-2-3p and preserved synchronous function with RNF144A-AS1.

Due to the positive correlation of RNF144A-AS1 and LOX with the hypoxia-related gene set, we then intend to verify the effect of hypoxia on RNF144A-AS1 expression. However, RNF144A-AS1 responded to hypoxia in a HIF-1α-independent way, suggesting another mechanistic explanation for the upregulation of RNF144A-AS1 under hypoxic conditions. In this regard, TGF-β signaling was proposed for its essential role in EMT and metastasis. In addition, several studies also suggested the interplay between TGF-β signaling and hypoxia [49, 50]. As indicated, the expression of RNF144A-AS1 and the other two genes were regulated by TGF-β1. Of note, the cross-regulation of TGF-β and LOX has already been found, especially in the fibrotic process [51, 52]. In GC, tumor cells in metastatic sites could secrete TGF- β1 to prompt the production of cancer-associated fibroblasts-derived LOX, which may explain the higher fold-change of LOX under the treatment of TGF-β1 [45]. Importantly, whether TGF-β1 stimulates the expression of RNF144A-AS1 alone or under the regulation of hypoxia should be further explored, and the effective ratio of TGF-β1-RNF144A-AS1-mediated upregulation of LOX also demand further study.

In conclusion, our study highlights the value of RNF144A-AS1 as a prognostic marker or therapeutic target of GC. RNF144A-AS1 predicted poor prognosis of patients with GC and was related to later-stage diseases such as tumor distant metastasis. Moreover, RNF144A-AS1 promoted GC metastasis, angiogenesis, and proliferation by competitively absorbing miR-30c-2-3p to release LOX. In addition, the expression of RNF144A-AS1 was further upregulated under hypoxic circumstances in a HIF-1α-independent way and was also stimulated by TGF-β1.

## Supplementary Information

**Additional file 1: Table S1.** Primers used for quantitative RT-PCR.

**Additional file 2: Table S2.** Sequences for siRNA and shRNA used in this study.

**Additional file 3: Table S3.** Associations between RNF144A-AS1 expression and clinicopathological features of gastric cancer.

**Additional file 4: Figure S1. **RNF144A-AS1 preserved non-coding feature. **A** Analysis of RNF144A-AS1 expression in tumor tissues with or without distant metastasis (n = 8 for each group). **B **The coding potential of RNF144A-AS1 was low from CAPT. **C** Prediction of coding potential to RNF144A-AS1 using ORF finder. *P < 0.05 by Student’s t-test unless otherwise specified.

**Additional file 5: Figure S2. **GSEA analysis of RNF144A-AS1 and the function of RNF144A-AS1 in GC cells. **A **GSEA analysis of RNF144A-AS1 based on gene expression data extracted from the TCGA database.** B **GSEA analysis of RNF144A-AS1 based on gene expression data extracted from the CCLE database. **C-D** Cell motility was examined in treated MKN45 (**C**) and AGS (**D**) cells by wound-healing assays. Scale bars = 200 um. **E** Cell growth of HGC27 cells treated by RNF144A-AS1 vector or control. Data were analyzed using two-way ANOVA. Error bars, mean ± SD from triplicate samples. **P < 0.01, ***P < 0.001 by Student’s t‐test unless otherwise specified.

**Additional file 6: Figure S3. **MiR-30c-2-3p was the target of RNF144A-AS1. **A** Heat map presentation of the expression level of predicted miRNAs in 41 paired GC tissues from the TCGA database. The red shades represent high expression, and the blue shades represent a low expression. **B** Kaplan-Meier curves of miR-30c according to TCGA database. *P*-value from log-rank test. **C** The expression of miR-139-3p in GC cells transfected with siRNAs against RNF144A-AS1 or control. **D** Pearson correlation analysis between RNF144A-AS1 and miR-30c-2-3p in 60 GC tissues. **E** Expression of miR-30c-2-3p in GC cells treated by miR-30c-2-3p inhibitor or control. **F** The protein level of EMT-related markers and VEGFA as indicated. **G** Cellular invasion of GC cells co-transfected with miRNA mimics and RNF144A-AS1 vector. Scale bars = 100 um. **H-I** Cell proliferation rate detected by CCK8 assays in MKN45 (**H**) and AGS (**I**) cells as indicated. Data were analyzed using two-way ANOVA. Error bars, mean ± SD from triplicate samples. *P < 0.05, **P < 0.01 by Student’s t‐test unless otherwise specified.

**Additional file 7: Figure S4. **LOX predicted poor prognosis of GC patients and was a target of miR-30c-2-3p. **A** qRT-PCR analysis of LOX in GC cells transfected with miR-30c-2-3p inhibitor or control. **B** Western blot analysis of LOX in GC cells transfected with miR-30c-2-3p inhibitor or control. **C** The expression of LOX in MKN45 cells co-transfected with miR-30c-2-3p inhibitor and RNF144A-AS1 siRNA. **D** Kaplan–Meier analysis showed the association between LOX expression and overall survival of gastric cancer patients with I-IV stages (left panel), III stage (middle panel), IV stage (right panel). *P*-value from log-rank test. **E** GSEA analysis of LOX based on gene expression data extracted from TCGA database. **F** GSEA analysis of LOX based on gene expression data extracted from the CCLE database. Error bars, mean ± SD from triplicate samples. **P < 0.01 by Student’s t‐test unless otherwise specified.

**Additional file 8: Figure S5.** The effect of TGF-β1 on LOX expression. **A **HIF-1α-specific siRNA efficiently inhibited the expression of HIF-1α in GC cells. **B-C **Enrichment plots of LOX with TGF-β signaling from the expression data of TCGA database (**B**) and CCLE database (**C**). **D** Western blot analysis of LOX expression in GC cells treated with recombinant human TGF-β1 or not.

## Data Availability

The datasets used and/or analyzed during the current study are available from the corresponding author on reasonable request.

## Abbreviations

GC: Gastric cancer
LOX: Lysyl oxidase
VEGFA: Vascular endothelial growth factor A
CD31: Platelet endothelial cell adhesion molecule-1
HIF-1α: Hypoxia-inducible factor 1α
TCGA: The Cancer Genome Atlas
CCLE: Cancer Cell Line Encyclopedia
GSEA: Gene Set Enrichment Analysis
NES: Normalized Enrichment Score
CeRNA: Competing endogenous RNA
LncRNA: Long noncoding RNA
ECM: Extracellular matrix
EMT: Epithelial–mesenchymal transition
HUVECs: Human umbilical vein endothelial cells
FISH: Fluorescence in situ hybridization
IHC: Immunohistochemistry
RIP: RNA immunoprecipitation
CM: Conditioned medium
TGF-β1: Transforming Growth Factor β1
