# Supplementary material for: RNF144A-AS1, a TGF-β1- and hypoxia-inducible gene that promotes tumor metastasis and proliferation via targeting the miR-30c-2-3p/LOX axis in gastric cancer
Source: Cell Biosci. 2021 Sep 28;11:177. doi: 10.1186/s13578-021-00689-z (PMC8480077; doi:10.1186/s13578-021-00689-z)
Supplement: Supplementary file 1 — Additional file 1: Table S1. Primers used for quantitative RT-PCR. [file 13578_2021_689_MOESM1_ESM.docx]

**Additional files: Additional tables**

**Additional file 1: Table S1** Primers used for quantitative RT-PCR.

| Gene | 5' to 3' |
| --- | --- |
| RNF144A-AS1 Forward | AGGATTCAGGGGATGCACAG |
| RNF144A-AS1 Reverse | TGGGCTGAAGATGAGACGTT |
| hsa-miR-30c-2-3p Forward | CTGGGAGAAGGCTGTTTACTCTAA |
| hsa-miR-30c-2-3p Universal reverse | GCGAGCACAGAATTAATACGAC |
| hsa-miR-139-3p Forward | TGGAGACGCGGCCCTGTTGGAGTAA |
| hsa-miR-139-3p Universal reverse | GCGAGCACAGAATTAATACGAC |
| LOX Forward | TGGGAATGGCACAGTTGTC |
| LOX Reverse | AAACTTGCTTTGTGGCCTTC |
| U6 Forward | CTCGCTTCGGCAGCACA |
| U6 Universal reverse | GCGAGCACAGAATTAATACGAC |
| GAPDH Forward | TGCACCACCAACTGCTTAGC |
| GAPDH Reverse | GGCATGGACTGTGGTCATGAG |
