# Supplementary material for: RNF144A-AS1, a TGF-β1- and hypoxia-inducible gene that promotes tumor metastasis and proliferation via targeting the miR-30c-2-3p/LOX axis in gastric cancer
Source: Cell Biosci. 2021 Sep 28;11:177. doi: 10.1186/s13578-021-00689-z (PMC8480077; doi:10.1186/s13578-021-00689-z)
Supplement: Supplementary file 2 — Additional file 2: Table S2. Sequences for siRNA and shRNA used in this study. [file 13578_2021_689_MOESM2_ESM.docx]

**Additional file 2: Table S2** Sequences for siRNA and shRNA used in this study.

| Gene | 5' to 3' |
| --- | --- |
| RNF144A-AS1 shRNA | TACAAAGGTGGCAAGATAAAT |
| RNF144A-AS1 siRNA 1# sense | GCUUUGACUUAGACUUCUAGC |
| RNF144A-AS1 siRNA 1# antisense | UAGAAGUCUAAGUCAAAGCUU |
| RNF144A-AS1 siRNA 2# sense | CUGUGAUGGUUAAUGUUAAGU |
| RNF144A-AS1 siRNA 2# antisense | UUAACAUUAACCAUCACAGGG |
| LOX siRNA sense | UAGAAGUCUAAGUCAAAGCUU |
| LOX siRNA antisense | UAGAAGUCUAAGUCAAAGCUU |
| LOX shRNA | GATTGATATTACAGATGTA |
| HIF-1α siRNA sense | CGAGGAAGAACUAUGAACATT |
| HIF-1α siRNA antisense | UGUUCAUAGUUCUUCCUCGTT |
| Dicer siRNA sense | TTTGTTGCGAGGCTGATTC |
