# Supplementary material for: RNF144A-AS1, a TGF-β1- and hypoxia-inducible gene that promotes tumor metastasis and proliferation via targeting the miR-30c-2-3p/LOX axis in gastric cancer
Source: Cell Biosci. 2021 Sep 28;11:177. doi: 10.1186/s13578-021-00689-z (PMC8480077; doi:10.1186/s13578-021-00689-z)
Supplement: Supplementary file 3 — Additional file 3: Table S3. Associations between RNF144A-AS1 expression and clinicopathological features of gastric cancer. [file 13578_2021_689_MOESM3_ESM.docx]

**Additional file 3: Table S3** Associations between RNF144A-AS1 expression and clinicopathological features of gastric cancer.

| characteristics | Group | Cases | |  |
| --- | --- | --- | --- | --- |
|  |  | low expression | high expression | *P* value |
| Age | < 60 | 18 | 14 |  |
|  | ≥ 60 | 16 | 12 | 0.944 |
| Gender | Male | 25 | 22 |  |
|  | Female | 9 | 4 | 0.302 |
| T stage | T1-T2 | 8 | 1 |  |
|  | T3-T4 | 26 | 25 | **0.034** |
| Lymph node metastasis | Absent (N0) | 13 | 3 |  |
|  | Present (N1-N3) | 21 | 20 | **0.015** |
| TNM stage | I-II | 19 | 5 |  |
|  | III-IV | 15 | 17 | **0.003** |
| Location | Cardia | 24 | 17 |  |
|  | Non-cardia | 10 | 9 | 0.563 |

Clinicopathological results were compared using Pearson χ^2^ tests.

The significant results are in bold.
