# Supplementary material for: RNF144A-AS1, a TGF-β1- and hypoxia-inducible gene that promotes tumor metastasis and proliferation via targeting the miR-30c-2-3p/LOX axis in gastric cancer
Source: Cell Biosci. 2021 Sep 28;11:177. doi: 10.1186/s13578-021-00689-z (PMC8480077; doi:10.1186/s13578-021-00689-z)
Supplement: Supplementary file 4 — Additional file 4: Figure S1. RNF144A-AS1 preserved non-coding feature. A Analysis of RNF144A-AS1 expression in tumor tissues with or without distant metastasis (n = 8 for each group). B The coding potential of RNF144A-AS1 was low from CAPT. C Prediction of coding potential to RNF144A-AS1 using ORF finder. *P < 0.05 by Student’s t-test unless otherwise specified. [file 13578_2021_689_MOESM4_ESM.pdf]

RNF144A-AS1, a TGF-β1- and hypoxia-inducible gene that promotes tumor metastasis and proliferation via targeting the miR-30c-2-3p/LOX axis in gastric cancer

Additional files: Supplementary Figures

Additional file 4: Figure S1

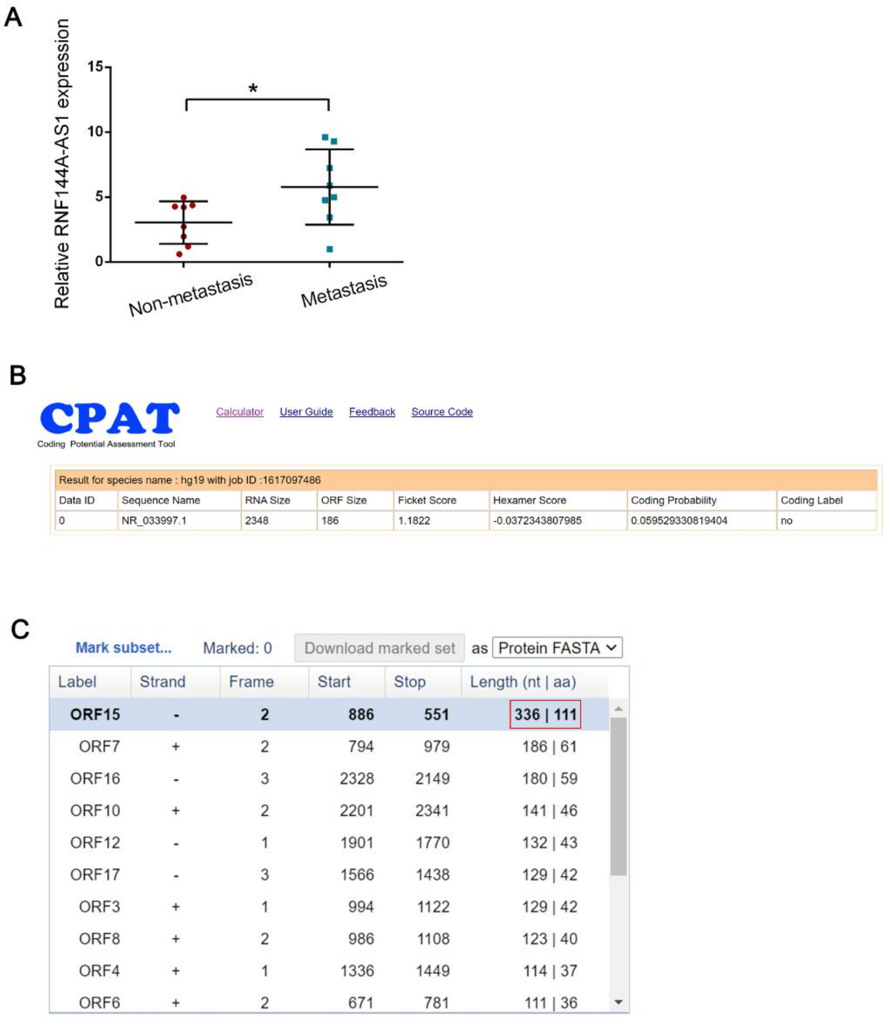

Figure S1 RNF144A-AS1 preserved non-coding feature. A Analysis of RNF144A-AS1 expression in tumor tissues with or without distant metastasis (n = 8 for each group). **B** The coding potential of RNF144A-AS1 was low from CAPT. **C** Prediction of coding potential to RNF144A-AS1 using ORF finder. **P* < 0.05 by Student's t-test unless otherwise specified.
