# Supplementary material for: RNF144A-AS1, a TGF-β1- and hypoxia-inducible gene that promotes tumor metastasis and proliferation via targeting the miR-30c-2-3p/LOX axis in gastric cancer
Source: Cell Biosci. 2021 Sep 28;11:177. doi: 10.1186/s13578-021-00689-z (PMC8480077; doi:10.1186/s13578-021-00689-z)
Supplement: Supplementary file 5 — Additional file 5: Figure S2. GSEA analysis of RNF144A-AS1 and the function of RNF144A-AS1 in GC cells. A GSEA analysis of RNF144A-AS1 based on gene expression data extracted from the TCGA database. B GSEA analysis of RNF144A-AS1 based on gene expression data extracted from the CCLE database. C-D Cell motility was examined in treated MKN45 (C) and AGS (D) cells by wound-healing assays. Scale bars = 200 um. E Cell growth of HGC27 cells treated by RNF144A-AS1 vector or control. Data were analyzed using two-way ANOVA. Error bars, mean ± SD from triplicate samples. **P < 0.01, ***P < 0.001 by Student’s t‐test unless otherwise specified. [file 13578_2021_689_MOESM5_ESM.pdf]

## Additional file 5: Figure S2

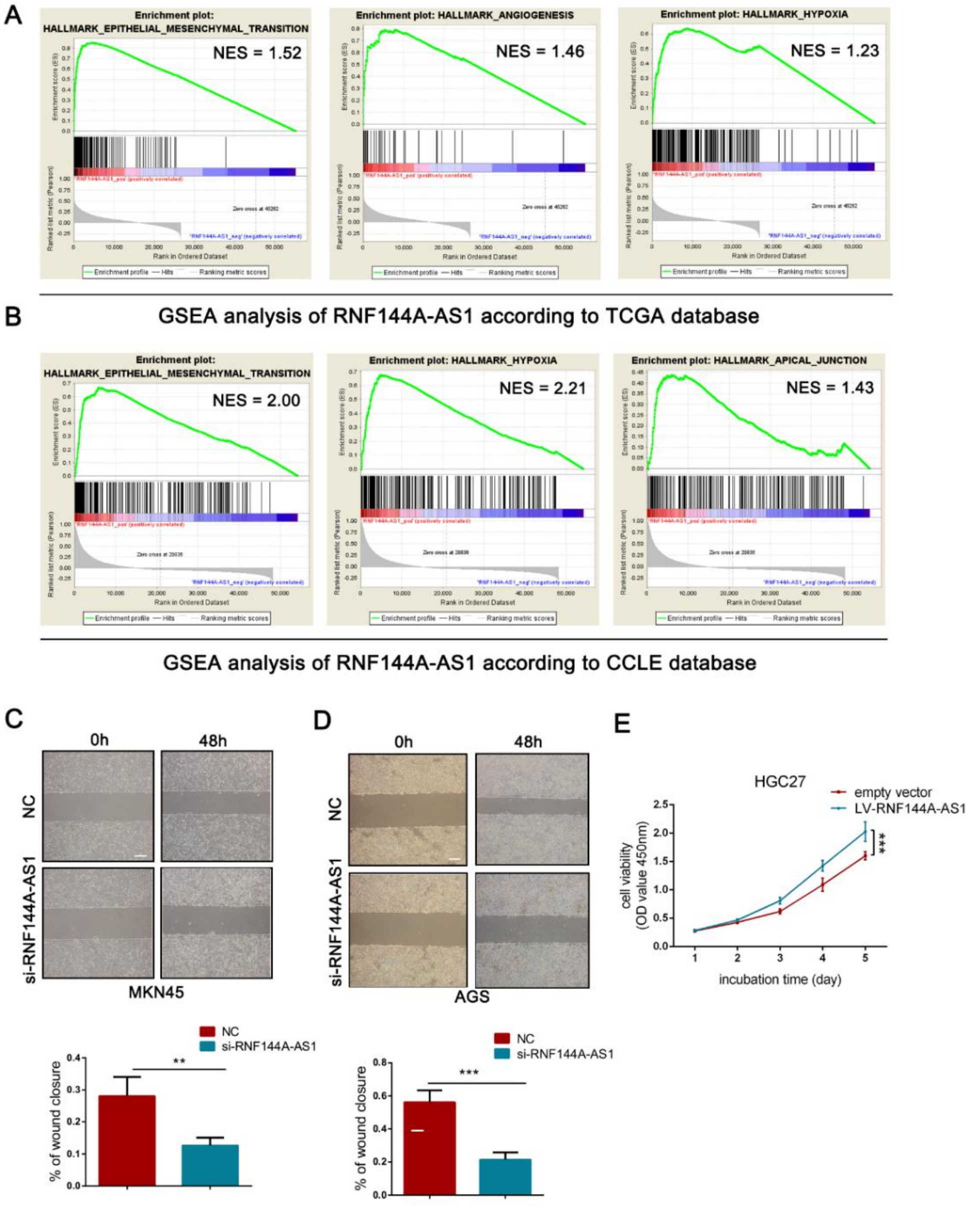


**Figure S2 GSEA analysis of RNF144A-AS1 and the function of RNF144A-AS1 in GC cells. A** GSEA analysis of RNF144A-AS1 based on gene expression data extracted from the TCGA database. **B** GSEA analysis of RNF144A-AS1 based on gene expression data extracted from the CCLE database. **C-D** Cell motility was

examined in treated MKN45 (**C**) and AGS (**D**) cells by wound-healing assays. Scale bars = 200 um. **E** Cell growth of HGC27 cells treated by RNF144A-AS1 vector or control. Data were analyzed using two-way ANOVA. Error bars, mean ± SD from triplicate samples. $^{**}P < 0.01$, $^{***}P < 0.001$ by Student's t-test unless otherwise specified.
