# Supplementary material for: RNF144A-AS1, a TGF-β1- and hypoxia-inducible gene that promotes tumor metastasis and proliferation via targeting the miR-30c-2-3p/LOX axis in gastric cancer
Source: Cell Biosci. 2021 Sep 28;11:177. doi: 10.1186/s13578-021-00689-z (PMC8480077; doi:10.1186/s13578-021-00689-z)
Supplement: Supplementary file 6 — Additional file 6: Figure S3. MiR-30c-2-3p was the target of RNF144A-AS1. A Heat map presentation of the expression level of predicted miRNAs in 41 paired GC tissues from the TCGA database. The red shades represent high expression, and the blue shades represent a low expression. B Kaplan-Meier curves of miR-30c according to TCGA database. P-value from log-rank test. C The expression of miR-139-3p in GC cells transfected with siRNAs against RNF144A-AS1 or control. D Pearson correlation analysis between RNF144A-AS1 and miR-30c-2-3p in 60 GC tissues. E Expression of miR-30c-2-3p in GC cells treated by miR-30c-2-3p inhibitor or control. F The protein level of EMT-related markers and VEGFA as indicated. G Cellular invasion of GC cells co-transfected with miRNA mimics and RNF144A-AS1 vector. Scale bars = 100 um. H-I Cell proliferation rate detected by CCK8 assays in MKN45 (H) and AGS (I) cells as indicated. Data were analyzed using two-way ANOVA. Error bars, mean ± SD from triplicate samples. *P < 0.05, **P < 0.01 by Student’s t‐test unless otherwise specified. [file 13578_2021_689_MOESM6_ESM.pdf]

Additional file 6: Figure S3

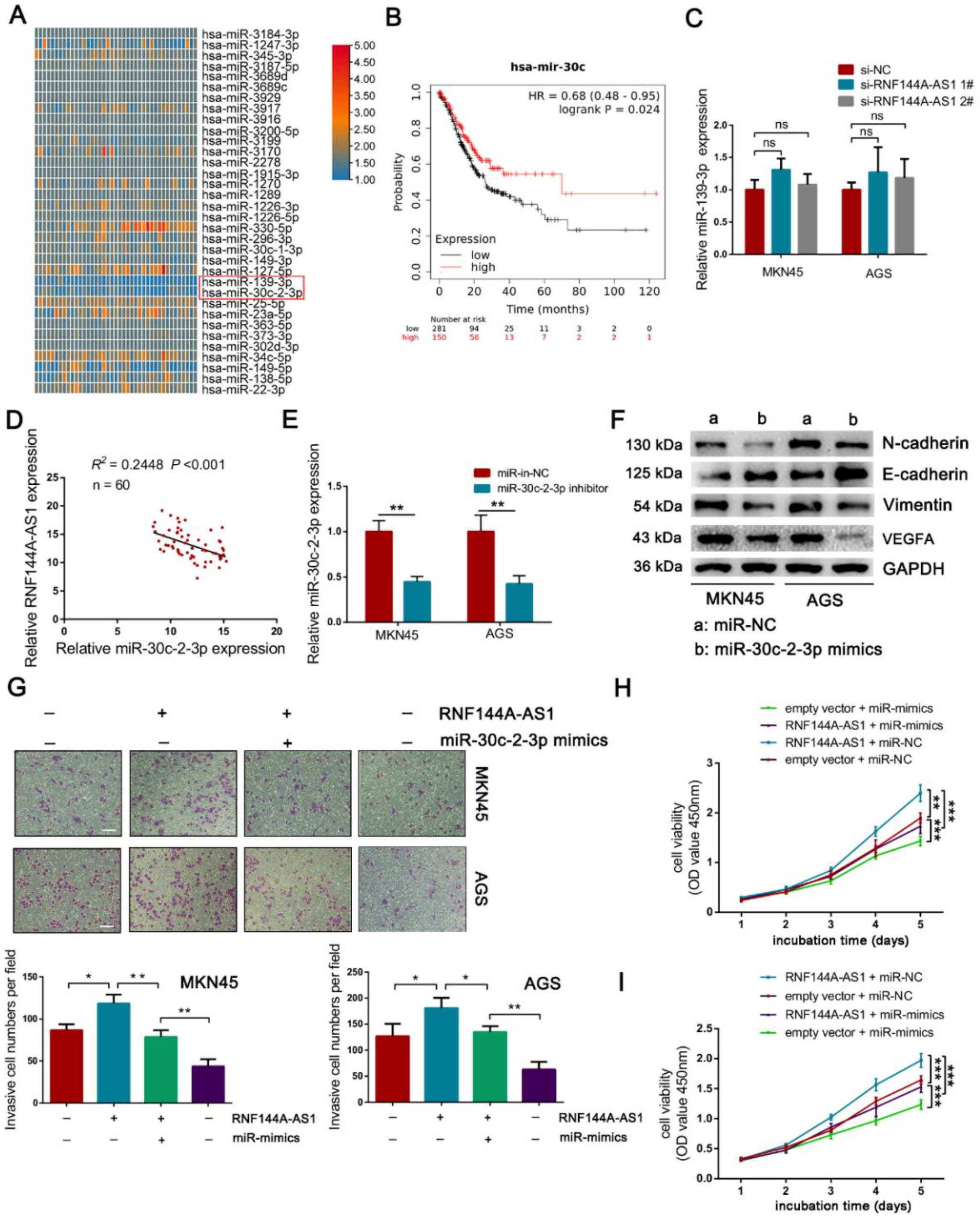

Figure S3 MiR-30c-2-3p was the target of RNF144A-AS1. A Heat map presentation of the expression level of predicted miRNAs in 41 paired GC tissues from the TCGA database. The red shades represent high expression, and the blue shades represent a low expression. **B** Kaplan-Meier curves of miR-30c according to

TCGA database. *P*-value from log-rank test. **C** The expression of miR-139-3p in GC cells transfected with siRNAs against RNF144A-AS1 or control. **D** Pearson correlation analysis between RNF144A-AS1 and miR-30c-2-3p in 60 GC tissues. **E** Expression of miR-30c-2-3p in GC cells treated by miR-30c-2-3p inhibitor or control. **F** The protein level of EMT-related markers and VEGFA as indicated. **G** Cellular invasion of GC cells co-transfected with miRNA mimics and RNF144A-AS1 vector. Scale bars = 100 um. **H-I** Cell proliferation rate detected by CCK8 assays in MKN45 (**H**) and AGS (**I**) cells as indicated. Data were analyzed using two-way ANOVA. Error bars, mean ± SD from triplicate samples. $^{*}P < 0.05$, $^{**}P < 0.01$ by Student's t-test unless otherwise specified.
