# Supplementary material for: RNF144A-AS1, a TGF-β1- and hypoxia-inducible gene that promotes tumor metastasis and proliferation via targeting the miR-30c-2-3p/LOX axis in gastric cancer
Source: Cell Biosci. 2021 Sep 28;11:177. doi: 10.1186/s13578-021-00689-z (PMC8480077; doi:10.1186/s13578-021-00689-z)
Supplement: Supplementary file 7 — Additional file 7: Figure S4. LOX predicted poor prognosis of GC patients and was a target of miR-30c-2-3p. A qRT-PCR analysis of LOX in GC cells transfected with miR-30c-2-3p inhibitor or control. B Western blot analysis of LOX in GC cells transfected with miR-30c-2-3p inhibitor or control. C The expression of LOX in MKN45 cells co-transfected with miR-30c-2-3p inhibitor and RNF144A-AS1 siRNA. D Kaplan–Meier analysis showed the association between LOX expression and overall survival of gastric cancer patients with I-IV stages (left panel), III stage (middle panel), IV stage (right panel). P-value from log-rank test. E GSEA analysis of LOX based on gene expression data extracted from TCGA database. F GSEA analysis of LOX based on gene expression data extracted from the CCLE database. Error bars, mean ± SD from triplicate samples. **P < 0.01 by Student’s t‐test unless otherwise specified. [file 13578_2021_689_MOESM7_ESM.pdf]

Additional file 7: Figure S4

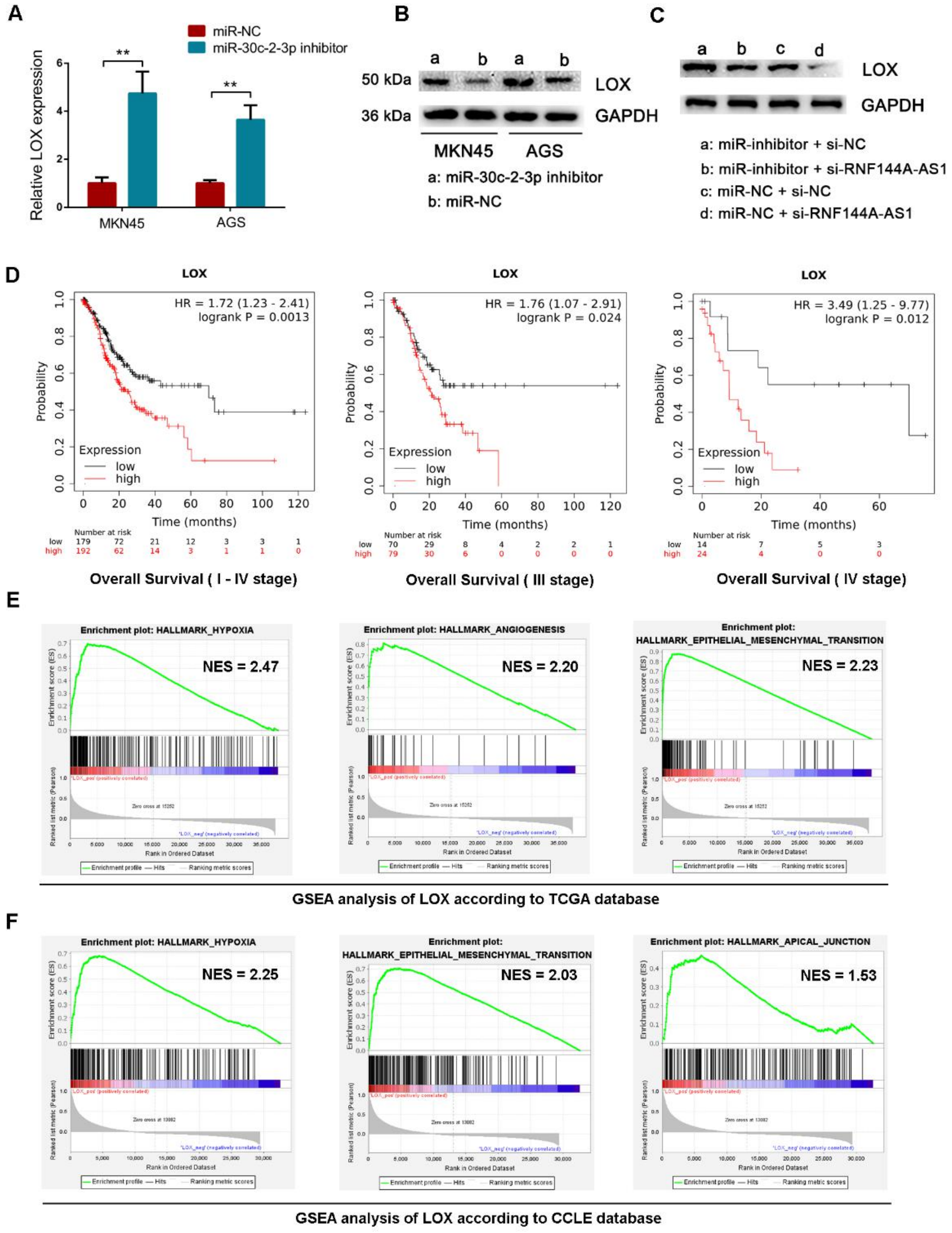

Figure S4 LOX predicted poor prognosis of GC patients and was a target of miR-30c-2-3p. **A** qRT-PCR analysis of LOX in GC cells transfected with miR-30c-2-3p inhibitor or control. **B** Western blot analysis of LOX in GC cells

transfected with miR-30c-2-3p inhibitor or control. **C** The expression of LOX in MKN45 cells co-transfected with miR-30c-2-3p inhibitor and RNF144A-AS1 siRNA. **D** Kaplan–Meier analysis showed the association between LOX expression and overall survival of gastric cancer patients with I-IV stages (left panel), III stage (middle panel), IV stage (right panel). P-value from log-rank test. **E** GSEA analysis of LOX based on gene expression data extracted from TCGA database. **F** GSEA analysis of LOX based on gene expression data extracted from the CCLE database. Error bars, mean ± SD from triplicate samples. $^{**}P < 0.01$ by Student's t-test unless otherwise specified.
