# Supplementary material for: RNF144A-AS1, a TGF-β1- and hypoxia-inducible gene that promotes tumor metastasis and proliferation via targeting the miR-30c-2-3p/LOX axis in gastric cancer
Source: Cell Biosci. 2021 Sep 28;11:177. doi: 10.1186/s13578-021-00689-z (PMC8480077; doi:10.1186/s13578-021-00689-z)
Supplement: Supplementary file 8 — Additional file 8: Figure S5. The effect of TGF-β1 on LOX expression. A HIF-1α-specific siRNA efficiently inhibited the expression of HIF-1α in GC cells. B-C Enrichment plots of LOX with TGF-β signaling from the expression data of TCGA database (B) and CCLE database (C). D Western blot analysis of LOX expression in GC cells treated with recombinant human TGF-β1 or not. [file 13578_2021_689_MOESM8_ESM.pdf]

Additional file 8: Figure S5

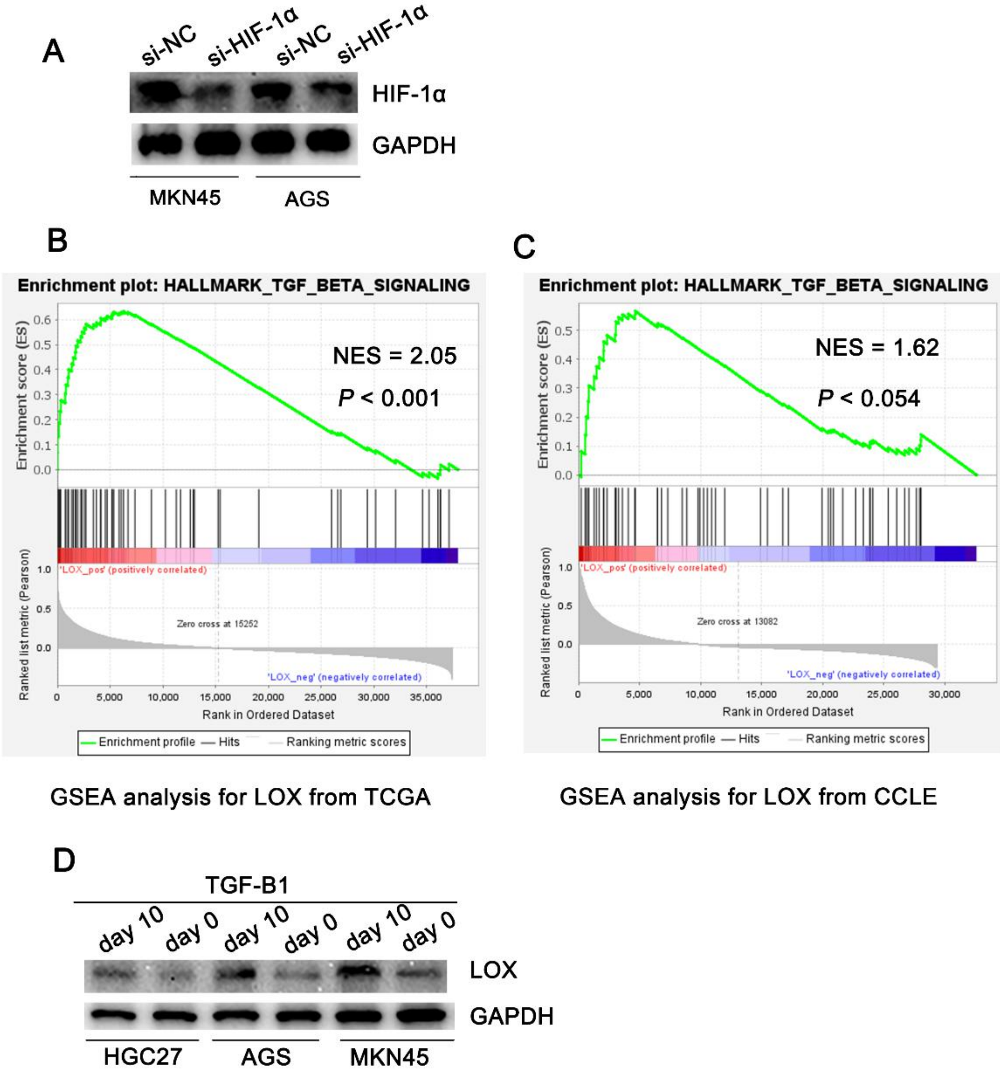

Figure S5 The effect of TGF-β1 on LOX expression. A HIF-1α-specific siRNA efficiently inhibited the expression of HIF-1α in GC cells. **B-C** Enrichment plots of LOX with TGF-β signaling from the expression data of TCGA database (**B**) and CCLE database (**C**). **D** Western blot analysis of LOX expression in GC cells treated with recombinant human TGF-β1 or not.
